# Simultaneous Optogenetics and Cellular Resolution Calcium Imaging During Active Behavior Using a Miniaturized Microscope

**DOI:** 10.3389/fnins.2018.00496

**Published:** 2018-07-24

**Authors:** Alice M. Stamatakis, Mike J. Schachter, Srishti Gulati, Kevin T. Zitelli, Sam Malanowski, Arash Tajik, Christopher Fritz, Mark Trulson, Stephani L. Otte

**Affiliations:** Inscopix Inc., Palo Alto, CA, United States

**Keywords:** optogenetics, calcium imaging, freely behaving, neurocircuits, GRIN lenses

## Abstract

The ability to precisely monitor and manipulate neural circuits is essential to understand the brain. Advancements over the last decade in optical techniques such as calcium imaging and optogenetics have empowered researchers to gain insight into brain function by systematically manipulating or monitoring defined neural circuits. Combining these cutting-edge techniques enables a more direct mechanism for ascribing neural dynamics to behavior. Here, we developed a miniaturized integrated microscope that allows for simultaneous optogenetic manipulation and cellular-resolution calcium imaging within the same field of view in freely behaving mice. The integrated microscope has two LEDs, one filtered with a 435–460 nm excitation filter for imaging green calcium indicators, and a second LED filtered with a 590–650 nm excitation filter for optogenetic modulation of red-shifted opsins. We developed and tested this technology to minimize biological and optical crosstalk. We observed insignificant amounts of biological and optical crosstalk with regards to the optogenetic LED affecting calcium imaging. We observed some amounts of residual crosstalk of the imaging light on optogenetic manipulation. Despite residual crosstalk, we have demonstrated the utility of this technology by probing the causal relationship between basolateral amygdala (BLA) -to- nucleus accumbens (NAc) circuit function, behavior, and network dynamics. Using this integrated microscope we were able to observe both a significant behavioral and cellular calcium response of the optogenetic modulation on the BLA-to-NAc circuit. This integrated strategy will allow for routine investigation of the causality of circuit manipulation on cellular-resolution network dynamics and behavior.

## Introduction

A fundamental goal of neuroscience is to understand how neural activity, adaptive behavior, and dysfunction emerge from complex electrical and chemical signaling within neural circuits. The ability to precisely measure and manipulate large-scale circuit activity during active behavior is critical to achieve this goal. Optical techniques such as optogenetics and calcium imaging are powerful tools to enable manipulation and recording of precisely defined neural circuits on physiologically relevant timescales. Optogenetics allows for the ability to establish a causal link between neural circuit activity and behavior by selectively manipulating distinct neural circuit elements while leaving surrounding elements unperturbed (Yizhar et al., 2011a). Innovations in bioreagents have enabled researchers to target neural circuits based on sophisticated features, such as their genetic profile, connectivity, or activity patterns, to determine whether precisely-defined circuits are necessary and/or sufficient for a behavior (Deisseroth, 2015). Parallel to advancements in optogenetics, calcium (Ca^2+^) imaging has undergone a methodological revolution. Improvements in biological reagents, optics such as gradient-index (GRIN) lenses, and experimental techniques have allowed for routine investigation of Ca^2+^ activity throughout the brain, from a subcellular to network level (Resendez and Stuber, 2015; Ji et al., 2016; Lin and Schnitzer, 2016). Thus, integrating Ca^2+^ imaging and optogenetics will permit direct access to the causal link between neural circuit dynamics, brain function, and behavior.

Several all-optical methods are currently being utilized to combine optogenetics with Ca^2+^ imaging *in vivo*. One method is to use a two-photon approach to combine optogenetics and Ca^2+^ imaging in awake head-fixed mice (Rickgauer et al., 2014; Packer et al., 2015; Baker et al., 2016; Carrillo-Reid et al., 2016). This technique combines two-photon Ca^2+^ imaging with two-photon cellular-resolution optogenetic manipulation, allowing for the ability to precisely activate defined subpopulations of neurons. Combining subcellular-resolution Ca^2+^ imaging with cellular-resolution optogenetic manipulation allows for a real-time investigation of the causal link between activity-defined populations of neurons and cellular and subcellular circuit dynamics. While these studies have demonstrated the feasibility of modulating subpopulations of neurons based on their activity responses, the sufficiency or necessity of these activity-dependent subpopulations for a specific behavior has yet to be determined. Finally, although improvements in virtual reality have allowed for an increasing range of experimental behaviors in a head-fixed mouse (Harvey et al., 2009; Aronov and Tank, 2014), the extent to which head-fixation impacts behavior or neuronal activity remains unclear. Thus, designing an optical system that is fully functional with complex freely-behaving models is necessary for enabling the full range of naturalistic animal behavior—an important element in understanding how endogenous neural activity leads to behavior.

Ca^2+^ imaging in freely-behaving mice has also been combined with simultaneous optogenetics using fiber photometry (Kim et al., 2016; Nieh et al., 2016). Using this all-optical approach, optogenetics is integrated with one-photon Ca^2+^ imaging through an optical fiber to allow for simultaneous optogenetics and Ca^2+^ imaging in the same field of view. While this method enables free behavior, neural activity analysis is restricted to an average bulk Ca^2+^ signal across the field of view, prohibiting the ability to investigate Ca^2+^ imaging at the single cell level. Recent studies investigating cellular-level Ca^2+^ dynamics have demonstrated heterogeneity in Ca^2+^ dynamics, even in a genetically homogenous population (Ziv et al., 2013; Jennings et al., 2015; Kitamura et al., 2015; Cox et al., 2016), underscoring the importance of cellular-resolution Ca^2+^ imaging.

Considering the limitations in current techniques to combine optogenetics with Ca^2+^ imaging, we engineered an integrated lightweight microscope that allows for simultaneous cellular-resolution Ca^2+^ imaging paired with full-field optogenetic manipulation in freely-behaving mice. We validated that the design of the imaging and optogenetic components of this microscope minimizes optical and biological crosstalk, although we observed some amount of residual biological crosstalk. We next imaged individual nucleus accumbens (NAc) neurons while stimulating reward-promoting basolateral amygdala (BLA) terminals to demonstrate the feasibility of performing these experiments in freely-behaving mice. Finally, we demonstrate that Ca^2+^ imaging data sets can be analyzed to extract significant effects of optogenetic manipulation on Ca^2+^ events and can be temporally aligned to behavioral changes *in vivo*.

## Methods

### Microscope configuration and LED control

The integrated microscope has two LEDs, one filtered with a 435–460 nm excitation filter (EX-LED) for imaging, and a second LED filtered with a 590–650 nm excitation filter (OG-LED) for optogenetics. The output from the EX-LED is controlled by a constant current source with the ability to drive up to 30 mA (2 mW/mm^2^ measured from the objective) with a step size of 7.6 μA. The OG-LED is controlled by a constant current source with the ability to drive up to 700 mA (10 mW/mm^2^ measured from the objective at 100% duty cycle, or 35 mW/mm^2^ at 25% duty cycle) with a step size of 15.2 μA.

**Figure 1:**
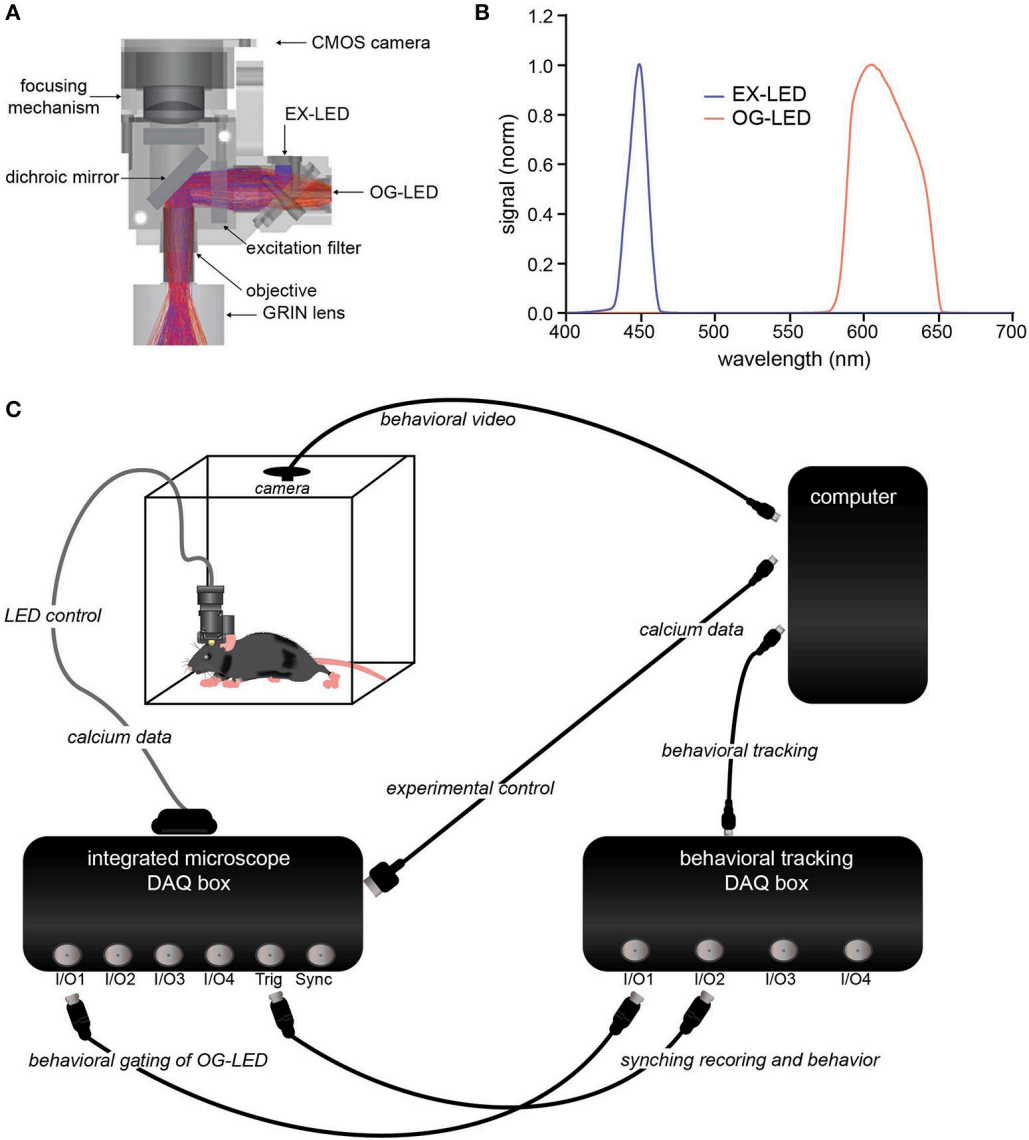
Integrated optogenetic and Ca^2+^ imaging system. **(A)** Cross-section schematic of the integrated optogenetic and Ca^2+^ imaging system detailing the filter design and the Ca^2+^ imaging LED (EX-LED) and optogenetic LED (OG-LED) light path. EX-LED rays (blue) and OG-LED rays (orange) are representative light paths measured using ray tracing. **(B)** EX-LED (blue) and OG-LED (orange) illumination spectra. Displayed spectra are normalized to equal peak spectral power. **(C)** Schematic of synchronized acquisition of integrated imaging and optogenetics with behavioral tracking.

The microscope has a maximum field of view of 1,440 × 1,080 pixels (900 × 650 μm). The microscope is capable of imaging at a 30 frames per second resolution at maximum field of view, and 50 frames per second at 50% of the field of view. The working distance of the microscope is 100–300 micrometers below the objective lens, with ~100 μm of adjustment below this plane available through the focusing element on the microscope tower (Figure 1A).

### Optical bench testing

The spectra of the miniaturized microscope (nVoke, Inscopix, Palo Alto, California) LED illumination were obtained by illuminating the end of a 400 micron 0.45 NA fiber optic (P400-2-UV-VIS, Ocean Optics, Dunedin, Florida) through an opal glass diffuser (opal glass diffuser, 43–717, Edmund Optics, Cupertino, California). The fiber optic directed the microscope output to a spectrograph with a cooled linear CCD sensor (QE65 Pro, Ocean Optics, Dunedin, Florida) with spectral resolution <2 nm. Displayed spectra are normalized to equal peak spectral power. Blank background testing were obtained by aiming the microscopes covered in black cloth into a dark room. Snapshots (50 ms exposure) were taken at OG-LED irradiance of 0 and 10 mW/mm^2^. This was repeated 4 times with 4 different microscopes. Measurements were taken from sections through the equators of the obtained images, averaged over 32 rows of pixels (46,080 total pixels). Temperature stability measurements were obtained by recording EX-LED light output over time using a light meter set to 448 nm (PM100D with 121C photodiode sensor, Thorlabs, Newton, New Jersey). To filter out OG-LED light, the light meter was covered with a 450/25 nm bandpass filter (Edmund Optics 86-653, Barrington New Jersey). Measurements were taken over a 30 min period with the EX-LED light set to 1 mW/mm^2^ for the entire session. OG-LED light was pulsed during minutes 5–25 of the recording (30 s off, 30 s on at continuous 10 mW/mm^2^, repeated). Data was acquired at 1 Hz.

### Animal subjects

All procedures were conducted in accordance with the Guide for the Care and Use of Laboratory Animals, as adopted by the National Institutes for Health, and with approval of the NASA Institutional Animal Care and Use Committee. Adult (25–30 g) C57BL/6J mice were group housed until surgery with *ad libitum* access to food and water. Mice were maintained on a reverse 12-h light cycle (lights off at 7:00 a.m.).

### Thermal bench testing

Thermal bench testings were adapted from Stujenske et al. (2015). Mice were deeply anesthetized with isofluorane. Brains were rapidly removed and placed in ice-cold PBS. Coronal slices (1 mm and 200 μM) were sliced on a Vibratome (VT-1200, Leica Microsystems, Wetzlar, Germany). Following slicing, brain slices were placed in room temperature PBS (~24°C) and were allowed to warm to ~24°C prior to recordings. To obtain temperature recordings, a thermocouple (IRCO-003, OMEGA Engineering, Inc., Stamford, Connecticut) was placed in between a 200 μm brain slice and two 1 mm brain slices stacked on top of each other. Brain slice stacks containing the thermocouple were placed on a standard microscope slide, covered in PBS, and cover slipped. The integrated microscope (nVoke, Inscopix, Palo Alto, California) was connected to a 4 mm GRIN lens (1 mm diameter, Part number 1050-002176, Inscopix, Palo Alto, California) using a modified microscope holder (ProView Kit, Inscopix, Palo Alto, California). The microscope holder allowed for an optimized distance between the objective and GRIN lens for imaging. The bottom of the 4 mm GRIN lens was positioned directly on top of the coverslip covering the 200 micron brain slice and thermocouple. Thus, the thermocouple was situated at the typical imaging plane of *in vivo* recordings. A 30 s baseline was established prior to OG-LED testing. Following the 30 s baseline test, brain slices were exposed to 2.5, 5, 10, or 35 (pulsed) mW/mm^2^ OG-LED light for 570 s. For the 35 mW/mm^2^ pulsing test, the duty cycle was set to 25%. For all other light deliveries, the duty cycle was set to 100% so that the slices received constant light. Slices recovered between each test until temperature returned to ~24°C. Testing was conducted on two technical and two biological replicates. Data was acquired at 1 Hz using a digital data logger (HH147U, OMEGA Engineering, Inc., Stamford, Connecticut).

### Viral constructs

Purified and concentrated adeno-associated virus serotype 5 coding for ChrimsonR-tdTomato under the synapsin promotor (AAV5-syn-ChrimsonR-tdT), eNpHR3.0-mCherry under the CamKIIa promotor (AAV5-CamKIIa-eNpHR3.0-mCherry), and Jaws-GFP under the CamKII promotor (AAV5-CamKII-Jaws-KGC-GFP-ER2) were packaged by the UNC Vector Core Facility at titers of ~10^12^ infectious units per mL. Purified and concentrated adeno-associated virus serotype DJ coding for GCaMP6s under the CamKIIa promotor (AAVDJ-CamKIIa-GCaMP6s) were packaged by the Stanford Vector Core at titers of 5.20 × 10^13^ infectious units per mL. To avoid overexpression and cell death, the GCaMP viral aliquots were diluted with sterile saline to a final titer of ~1 × 10^13^.

### Stereotaxic surgery

For all surgeries, mice were anesthetized with 1.5–2.0% isofluorane mixed with 1 L per min of oxygen and placed in a stereotaxic frame (Kopf Instruments, Tujunga, California). Body temperature was maintained at 37°C with a heating pad (40-90-2-07, FHC, Bowdoin, Maine). Mice received subcutaneous injections of ketoprofen (2.5 mg/kg) and carprofen (2.5 mg/kg).

Mice used for somatic electrophysiological experiments were unilaterally microinjected with 0.5 μL of AAV5-syn-ChrimsonR-tdT, AAV5-CamKII-Jaws-KGC-GFP-ER2, or AAV5-CamKIIa-eNpHR3.0-mCherry into the somatosensory cortex at two injection sites (coordinates from Bregma: −1.0 posterior, −1.5 lateral, −0.65 and −0.3 ventral). Mice used for synaptic terminal electrophysiological experiments were microinjected with 0.5 μL of AAV5-syn-ChrimsonR-tdTom into the BLA (coordinates from Bregma: −1.6 posterior, −3.3 lateral, −4.6 ventral). Mice used for simultaneous optogenetic and imaging experiments were microinjected with 0.5 μL of AAV5-syn-ChrimsonR-tdTom into the BLA. Control mice received no injections into the BLA.

Four weeks following the BLA injections, to allow for sufficient opsin trafficking, control and experimental mice underwent a surgery to enable imaging of NAc neurons. A cranionomy above the NAc (coordinates from bregma: +1.1 anterior, −1.5 lateral), was performed and dura was carefully removed. Three hundred to five hundred micrometers of tissue was aspirated using a 30-gauge blunt needle attached to a vacuum pump to allow for an entry path for the GRIN lens. Saline was continuously applied during the aspiration to avoid drying of the tissue. Bleeding was controlled by sterile saline-soaked gelfoam. Following aspiration, mice were microinjected with 0.5 μL of AAVDJ-CamKIIa-GCaMP6s into the NAc (coordinates from bregma: +1.1 anterior, −1.5 lateral, −4.5 ventral). Immediately following injection, mice were implanted with a cuffed 7 mm long GRIN lens (coordinates from bregma: +1.1 anterior, −1.5 lateral, −4.3 ventral; Part number 1050-002208, Inscopix, Palo Alto, California) to enable optical access to the NAc and stimulation of BLA terminals (Stuber et al., 2011). For *in vivo* OG-LED crosstalk experiments, a separate group of mice were implanted with a cuffed 4 mm GRIN lens above the hippocampus (coordinates from bregma: −2.0 posterior, −1.35 lateral, −1.45 ventral; Part number 1050-002202, Inscopix, Palo Alto, California), or dorsal striatum (coordinates from bregma: +1.0 anterior, −1.5 lateral, −2.4 ventral; Part number 1050-002202, Inscopix, Palo Alto, California). During implantation, lenses were held in place and slowly lowered using a ProView kit (1050-002334, Inscopix, Palo Alto, California) attached to a stereotax arm (Kopf Instruments, Tujunga, California). The portion of the lens extending above the surface of the skull (~2 mm) was fixed with metabond (Parkell, Edgewood, New York) and anchored by 2 skull screws (19010-10, Fine Science Tools, Foster City, California). A silicon elastomer (Kwik-Kast, World Precision Instruments) was applied to the top of the protective lens cuff to protect the top of the lens from debris or damage.

Three to four weeks following the lens implantation surgery, mice were equipped with a baseplate docking station to support the integrated microscope on top of the mouse's head (BPL-2, Inscopix, Palo Alto, California). The silicone elastomer was detached from the lens cuff and the top of the lens was cleaned using ddH_2_0 and lens paper (Thorlabs, Newton, New Jersey). An adjustable microscope gripper (1050-002199, Inscopix, Palo Alto, California) positioned the integrated microscope with an attached baseplate above the top of the implanted lens. The lens and objective of the microscope were aligned such that the face of both lenses were parallel to each other. Using a manual stereotax arm (Kopf Instruments, Tujunga, California), the microscope gripper was lowered until the field of view was in focus, as determined by defined landmarks, such as cells or blood vessels. The baseplate was then adhered to the mouse's head using metabond (Parkell, Edgewood, New York). The entire head-cap was painted with black nail polish to minimize light leakage. A baseplate cover (BPC-2, Inscopix, Palo Alto, California) was then secured on the baseplate to protect the lens prior to imaging experiments.

### *In vivo* OG-LED crosstalk

For mice not expressing GCaMP indicator, snapshots (50 ms exposure) were taken at OG-LED powers of 0 and 10 mW/mm^2^ (100% duty cycle). This was repeated 3 times in 2 mice. Measurements were taken from sections through the equators of the obtained images, averaged over 32 rows of pixels (46,080 total pixels).

For mice expressing GCaMP, 8 min of recording was captured (15 frames per second, 66.7 ms exposure, 1.0 mW/mm^2^ EX-LED). After 3 min of recording, 3 bouts of 30 s of OG-LED irradiation (10 mW/mm^2^, 100% duty cycle) was delivered, with a 30 s off period in between. Raw fluorescent counts from *n* = 40 GCaMP cellular region of interests (ROIs) were averaged from the 3 OG-LED on time periods (450 frames per bout, 1,350 frames total), and were compared to a 90 s period of the baseline (1,350 frames total).

### Histology and confocal imaging

Mice were deeply anesthetized with isofluorane and were perfused with phosphate-buffered saline (PBS) followed by 4% paraformaldehyde in PBS. Forty micrometers of brain sections containing the BLA and NAc were mounted with Vectashield containing DAPI (Fisher Scientific, Waltham, Massachusetts). Captured and tiled images of mounted sections were captured on a Zeiss 710 confocal microscope using a 10X or 20X objective. For determination of lens and virus placements, tissue was imaged at 10X on an upright fluorescent microscope.

### Slice preparation for patch-clamp electrophysiology

Mice were deeply anesthetized with isofluorane. Brains were rapidly removed and placed in a modified artificial cerebrospinal fluid at ~0°C containing (in mM): 225 sucrose, 119 NaCl, 1.0 NaH_2_PO_4_, 4.9 MgCl_2_, 0.1 CaCl_2_, 26.2 NaHCO_3_, 1.25 glucose. Coronal slices containing the somatosensory cortex or NAc (200 μM) were sliced on a Vibratome (VT-1200, Leica Microsystems, Wetzlar, Germany). Following slicing, brain slices were placed in a holding chamber containing a bicarbonate-buffered solution saturated with 95% O_2_ and 5% CO_2_ containing (in mM): 119 NaCl, 2.5 KCl, 1.0 NaH_2_PO_4_, 1.3 MgCl, 2.5 CaCl_2_, 26.2 NaHCO_3_, 11 glucose (at 23–24°C). Slice were allowed to recover for at least 30 min before being placed in the recording chamber and superfused with the bicarbonate-buffered solution.

### Patch-clamp electrophysiology

For voltage-clamp recordings, patch electrodes (3–5 MΩ) were back-filled with a cesium methanesulfonic acid solution containing (in mM): 117 Cs methanesulfonic acid, 20 HEPES, 0.4 EGTA, 2.8 NaCl, 5 TEA, 2 ATP, 0.2 GTP. For current-clamp recordings, patch electrodes (3–5 MΩ) were back-filled with a potassium gluconate solution containing (in mM): 130 K-gluconate, 10 KCl, 10 HEPES, 10 EGTA, 2 MgCl_2_, 2ATP, 0.2 GTP. pH = 7.35, 270–285 mOsm for all internal solutions. Cells were visualized using infrared differential contrast and fluorescence microscopy. Whole-cell recordings were made using a MultiClamp 700B amplifier (Molecular Devices, Sunnyvale, California). For optical photostimulation, blue light (pE-4000, 460 nm, CoolLED, Andover, UK) was filtered with the EX-LED excitation filter (nVoke, Inscopix, Palo Alto, California) and delivered through a 40X objective. Orange light (pE-4000, 550 and 635 nm at a 100:15 ratio, CoolLED, Andover, UK) was filtered with the OG-LED excitation filter (nVoke, Inscopix, Palo Alto, California) and delivered through a 40X objective. Series resistance (15–25 Ω) and/or input resistance was monitored online using a 5 mV hyperpolarizing step delivered between stimulation sweeps. All data was filtered at 2 kHz, digitized at 5 kHz, and collecting using pClamp10 software (Molecular Devices, Sunnyvale, California).

For ChrimsonR synaptic terminal experiments, OG-LED light pulses (5 ms, 2 mW/mm^2^) or EX-LED light pulses (5 ms, 2 mW/mm^2^) were delivered every 20 s to evoke postsynaptic currents. EPSC amplitudes were calculated by measuring the peak current from the average response from >5 sweeps. For the ChrimsonR synaptic terminal timecourse experiment, OG-LED light pulses (5 ms pulses, 10 Hz, 13 mW/mm^2^) were delivered every 20 s for 5 sweeps to evoke postsynaptic currents. Pulse trains were repeated every 5 min for a total of 30 min of recording. In a subset of neurons, EX-LED (20 min, 0.5 mW/mm^2^) was delivered between minutes 5 and 25. EPSC amplitudes were calculated by measuring the peak current from the average response from 5 sweeps. In a few cases, BLA terminal stimulation elicited spikes. As necessary, EPSC amplitude was estimated by generating an exponential curve fit to the latter 300–400 ms of the mean EPSC trace. EPSC amplitude was estimated as the intercept point of the extrapolated curve fit and the rising phase of the EPSC trace.

For all somatic recordings, OG-LED light pulses (500 ms, 0.2–2.0 mW/mm^2^) or EX-LED (500 ms, 0.2–2.0 mW/mm^2^) were delivered every 20 s to evoke postsynaptic currents, or action potential firing. EPSC amplitudes were calculated by measuring the peak current from the average response from 5 sweeps.

### Real-time place preference

At least 24 h after baseplate installation mice were habituated daily to the integrated microscope (nVoke, Inscopix, Palo Alto, California) in their home-cages. Prior to all habituation and behavioral sessions the integrated microscope was attached to the baseplate (Part number 1050-002192, Inscopix, Palo Alto, California) and fixed in place by the baseplate set screw. Habituation sessions lasted between 5 and 30 min for at least 3 days. Following habituation, mice were placed in a 30 × 30 cm open field box (Phenotyper Box, Noldus, Wageningen, The Netherlands). Mice received two 30 min habituation sessions separated by at least 24 h in the box in order to habituate the mice to the box. At least 24 h following the second habituation session, mice received at least 2 days of real-time place preference (RTPP). Mice received at least one counterbalanced 30 min session each of an EX-LED RTPP and OG-LED RTPP. Minutes 0–5 and 25–30 were used to establish a pre- and post-RTPP baseline. During the RTPP portion of the session (minutes 5–25) one counterbalanced side of the chamber was assigned as the LED-paired side. Each time the mouse crossed to the LED-paired side of the chamber, LED stimulation was delivered until the mouse crossed back into the non-stimulation side. For the OG-LED RTPP mice received 1–2 s of 20 Hz OG-LED (5 ms pulse duration, 5–35 mW/mm^2^ measured at the objective) every 3–4 s. These optogenetic parameters were chosen prior to the RTPP session (>24 h), by visually inspecting the effect of the OG-LED on calcium responses. The OG-LED parameters (pulse train duration and OG-LED light levels) were gradually ramped up until a robust postsynaptic effect was observed (increase in calcium activity visualized under df/f). For the EX-LED RTPP mice received 1–2 s of EX-LED (constant, 0.5–1.2 mW/mm^2^ measured at the objective to mimic recording parameters) every 3–4 s. Behavior was tracked during the entire session using the Ethovision XT software (Noldus, Wageningen, The Netherlands). Percent time spent on the LED-paired side, velocity, and distance traveled was recorded via the Ethovision XT software. During the OG-LED RTPP Ca^2+^ imaging was recorded during the entire 30 min.

### Freely behaving Ca^2+^ imaging

An integrated microscope (nVoke, Inscopix, Palo Alto, California) was used to image GCaMP6s in NAc neurons through an implanted 7 mm GRIN lens (Part number 1050-002179, Inscopix, Palo Alto, California). Grayscale HDF5 images (12 bit) were acquired using nVoke Acquisition Software (version 2.1, Inscopix, Palo Alto, California) at 15 frames per second with an average exposure time of 66.7 ms. The analog gain on the image sensor was set between 1.5 and 3.0, while the EX-LED was maintained within 0.5–1.2 mW/mm^2^ (measured from the bottom of the objective). Ca^2+^ imaging, optogenetic activation, and behavior was time-stamped and synchronized.

### Image processing

All image processing was performed using Mosaic Software (version 1.2, Inscopix, Palo Alto, California). First, a spatial downsampling of 2 was applied to each movie to reduce the file size and decrease the processing time. To enable parallelization, each movie was broken into 30 s segments. Background subtraction was performed on each segment using a spatial bandpassing Gaussian filter. Each segment was motion corrected with TurboReg (Thévenaz et al., 1998), using the mean image over time as the reference image. Any recording session that exhibited translational motion of more than 20 pixels was discarded from analysis. In a subset of the recordings, motion artifacts <20 pixels that were not corrected by translational motion correction were observed. In these recordings, we occasionally observed cells that temporarily moved outside of its ROI during an OG-LED pulse train. This could reduce the value of the ROI trace, decreasing the likelihood that the cell would be considered OG-responsive, producing a false negative. We observed no cases in which an OG-responsive cell moved into the ROI of a non-OG responsive cell (creating a false positive).

The background subtracted, motion corrected segments were then re-combined into a single movie and cropped to remove motion correction edge effects. The mean image over the entire movie was computed to produce an F0 image, and the DF/F was computed using F0.

### Cell identification

To identify cells, we applied Principal Component Analysis/Independent Component Analysis (PCA-ICA) to the DF/F movie (Mukamel et al., 2009). Cell activity was observed in the movie to be correlated, especially during OG stimulation. PCA-ICA can exhibit issues with correlated data, so we developed a process to convert the unmixing images produced by the algorithm to ROIs. First, we thresholded each unmixing image, leaving only the positive pixels. Then we re-standardized the image, dividing by the pixel intensity standard deviation. Another threshold was applied which zeroed out any pixels below 10 SDs. This method usually produced spatially compact ROIs. If not, the connected components of the thresholded image were identified, and if there was more than one, only the largest connected component was retained. After conversion to ROIs, traces were computed by temporal convolution of the ROI with the DF/F movie. An automatic merging procedure was run which combined ROIs that were in close spatial proximity and whose traces exhibited a correlation coefficient of >0.60. Traces were re-computed for the merged ROIs. For some movies, ROIs were manually dropped if they falsely identified cells, or created for cells that were not automatically identified.

### GCaMP noise

The GCaMP noise level was measured in each neuron from spatially bandpassed movies where the mean intensity over pixels was subtracted from each frame. To measure the noise level, the ROI for each neuron, previously obtained through PCA-ICA, was thresholded such that all pixels in the ROI < 0.75 times the maximum pixel intensity were set to zero. The thresholded ROI was then applied to the spatially bandpassed movie to produce a trace. The median absolute deviation of the trace was measured, in units of counts, and was used as the GCaMP noise level for that neuron.

### Detection of OG-stimulated cells

An encoder analysis was performed to determine which cells were responsive to OG stimulation. The encoder is a modified temporal receptive field (Theunissen et al., 2000) that translated the recent history of OG stimulation into a prediction of DF/F activity for a given cell. First, the OG stimulation onset times were transformed into a binary temporal sequence, where a 1 denoted the onset of 1 s of 20 Hz stimulation, and left to be zero otherwise. A linear filter encoder was then fit to predict the value of a trace at time t from the preceding ~6 s of OG onsets. The encoder was fit using cross validated Ridge regression. Ten different ridge parameters were tried, logarithmically spaced from 10^−2^ to 10^5^. For each ridge parameter, 10-fold cross validation was performed on the dataset. The R2 was computed on each holdout set, and the average *R*^2^ was computed across holdout sets. The ridge parameter that produced the highest average *R*^2^ was chosen as optimal, and the average *R*^2^ for that parameter was used as the overall performance metric for that cell. Cells that exhibited an *R*^2^ ≥ 0.05 and an event rate >0.01 Hz were considered “OG-stimulated.”

### Statistical analysis

Results are expressed as mean ± SEM. All analysis were performed using MATLAB and Graphpad Prism. Data was analyzed using paired *t-*tests, unpaired *t-*tests, and repeated-measures one-way ANOVAs where applicable. Significance levels: ^*^p < 0.05 ^**^p < 0.01. D'Agostino-Pearson or Shapiro–Wilk (for data sets with lower sample sizes) were used to check for normality prior to any significance test. All data demonstrated normality.

## Results

### Microscope design

We engineered an integrated imaging and optogenetic microscope from existing technology that allows for freely-behaving cellular-resolution Ca^2+^ imaging in rodents (Ghosh et al., 2011). To integrate simultaneous optogenetic manipulation, we made a number of additions and modifications (Figure 1A). First, we utilized a LED filtered with a 435–460 nm excitation filter (EX-LED; Figures 1A,B) for excitation of green Ca^2+^ indicators, such as GCaMP, a genetically encoded Ca^2+^ indicator (Chen et al., 2013). This blue-shifted LED was selected to minimize cross activation of red-shifted opsins, which have a broad action spectra (Gradinaru et al., 2010; Chuong et al., 2014; Klapoetke et al., 2014).

Next, we chose a red-shifted LED and excitation filter combination that would allow for maximal activation of red-shifted opsins, while minimizing light leakage into the imaging channel. We utilized a LED with a 590–650 nm excitation filter (OG-LED; Figures 1A,B). This LED allows for activation of a significant portion of the red-shifted opsins action curve (Gradinaru et al., 2010; Chuong et al., 2014; Klapoetke et al., 2014), while ensuring enough of a spectral guard-band is present to minimize the probability of stray light leaking to the imaging sensor. The light from both LEDs pass through a GRIN objective lens housed on the microscope (Figure 1A). The objective can then be paired with relay GRIN lenses of various sizes, enabling optical access throughout the brain, including cortex, hippocampus, striatum, and hypothalamus (Resendez et al., 2016).

To enable integration with behavior, we designed a data acquisition (DAQ) box containing multiple digital and analog input and output ports (Figure 1C). This allows for precise integration and temporal alignment between Ca^2+^ data and various experimental modalities, such as behavioral tracking, EEG, or electrophysiology. In addition, this DAQ box enables the OG-LED to be gated by signals from external devices, such as TTL pulses generated by a discrete animal behavior, which is a common optogenetic experimental design (Adamantidis et al., 2011; Stamatakis and Stuber, 2012; Matthews et al., 2016).

### OG-LED crosstalk

First, we sought to test whether delivering OG-LED light pulses would interfere with Ca^2+^ imaging. OG-LED interference could arise from a number of sources, including the imaging sensor detecting stray OG-LED light, OG-LED-induced autofluorescence excitation, or excitation of GCaMP by the OG-LED.

**Figure 2:**
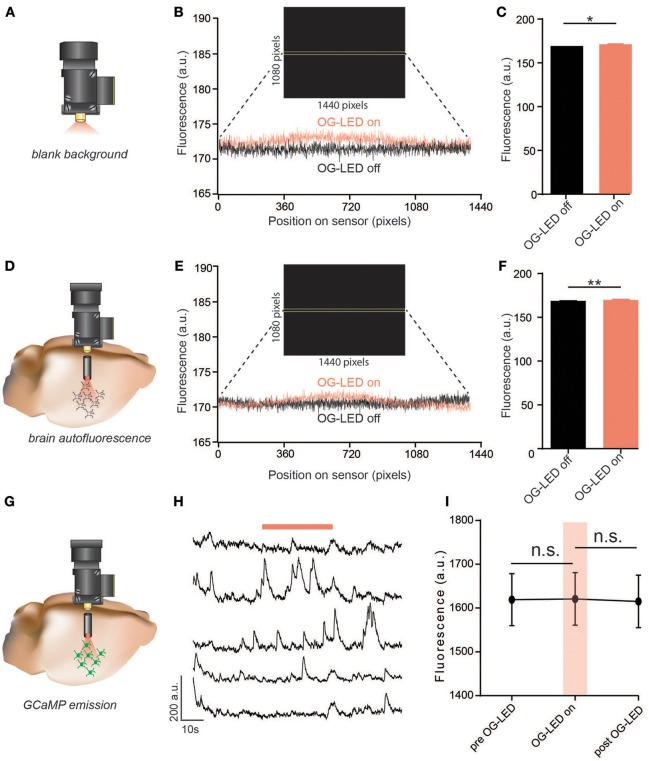
Exposure to OG-LED results in minimal fluorescence emission from microscope, lens, brain tissue, or GCaMP. **(A)** Schematic of *ex-vivo* OG-LED optical crosstalk measurements. The microscope was aimed into a black box in a dark room and fluorescence was measured during snapshots of OG-LED off (0 mW/mm^2^) and OG-LED on (10 mW/mm^2^) periods (50 ms exposure). This was repeated 4 times with 4 different microscopes. **(B)** Example fluorescent counts measured across the sensor through the equators of the obtained images, (OG-LED off and OG-LED on) averaged over 32 rows of pixels (46,080 total pixels). Inset: example frame captured during an OG-LED on snapshot, with yellow bars highlighting where the counts were averaged across the sensor. **(C)** OG-LED increased the average fluorescence detected by the sensor 2.06 ± 0.43 counts (*n* = 4 microscopes, paired *t*-test, *p* < 0.01). **(D)** Schematic of *in-vivo* measurements of OG-LED-induced emission of brain tissue autofluorescence. The integrated microscope was interfaced with a GRIN lens and changes in fluorescence were measured during snapshots of OG-LED off (0 mW/mm^2^) and OG-LED on (10 mW/mm^2^) periods from a mouse that is not expressing any foreign fluorescent protein. **(E)** Example fluorescent counts measured across the sensor through the equators of the obtained images, (OG-LED off and OG-LED on) averaged over 32 rows of pixels (46,080 total pixels). Inset: example frame captured during an OG-LED on snapshot, with yellow bars highlighting where the counts were averaged across the sensor. **(F)** OG-LED increased the average fluorescence detected by the sensor 2.54 ± 0.14 counts (*n* = 6 recordings from *n* = 2 mice, paired *t*-test, *p* < 0.01). **(G)** Schematic of *in-vivo* measurements of changes in GCaMP emission during exposure to OG-LED light (10 mW/mm^2^). Experiments were conducted by interfacing the integrated microscope with a GRIN lens measuring fluorescence changes in GCaMP expressing neurons in a mouse brain. **(H)** Representative Ca^2+^ traces of individual neurons. Orange bar represents OG-LED on period. **(I)** OG-LED off, OG-LED on, OG-LED off: before, during, after an OG-LED light pulse (30 s each, average of 3 trials). The average fluorescent counts did not significantly change during the 30 s OG-LED pulse (*n* = 40 cells from *n* = 2 mice, repeated-measures one-way ANOVA followed by Bonferroni multiple pairwise comparisons, pre vs. on, *p* = 0.99; post vs. on, *p* = 0.09). All error bars are SEM. *p < 0.05; **p < 0.01.

To test whether the design of the microscope, LEDs, or filters resulted in any OG-LED light-leakage that could be detected by the sensor, we measured changes in fluorescence counts (proportional to the # of photons per pixel) at the sensor in response to 10 mW/mm^2^ (the maximum irradiance enabled at a 100% duty cycle; Figure 2A). Across four microscopes tested, we measured an increase in 2.06 ± 0.43 counts at the sensor (Figures 2B,C). While this effect was significant (*t* = 4.77, *p* < 0.01), it is well below the average noise we observe in extracted GCaMP traces (GCaMP noise = 6.15 ± 0.15 counts, *n* = 1,060 extracted ROI traces from *n* = 15 mice), and thus is not likely to affect imaging acquisition, analysis, or data interpretation. These data also indicate that if the OG-LED is exciting any inorganic autofluorescence in the microscope or GRIN lens, it is being filtered out by the GCaMP emission filter.

Next, we investigated whether the OG-LED excited autofluorescence in brain tissue that is not filtered out by the GCaMP emission filter. A number of biological substances in the brain, such as lipofuscins and hemosiderin, are autofluorescent (Billinton and Knight, 2001; Duong and Han, 2013). If not filtered out appropriately, autofluorescence emission induced by OG-LED light could contribute to fluctuating fluorescent signals unrelated to Ca^2+^ changes, potentially confounding data analysis. To test this, we implanted a 4 mm GRIN lenses (1 mm diameter) into the dorsal striatum or hippocampus of mice that were not injected with any opsin or indicator (Figure 2D). As the mouse was freely behaving, we measured changes in fluorescence counts at the sensor in response to 10 mW/mm^2^ OG-LED light pulses. Similar to the blank background testing (Figures 2A–C), we found that the OG-LED significantly increased the fluorescent count value 2.54 ± 0.14 counts (*t* = 18, *p* < 0.01, Figures 2E,F), but as stated above, this is below the level of the typical noise in GCaMP traces. This data suggests that any autofluorescence or scattered OG-LED light generated in the brain tissue is blocked by the emission filter and thus should not influence Ca^2+^ imaging or analysis.

The excitation spectrum of GCaMPs strongly indicate that the 590–650 nm OG-LED should not excite the indicators in the absence of any opsin activation (Chen et al., 2013). However, to directly test this, we expressed GCaMP in the NAc and implanted 7 mm GRIN lenses above the NAc (0.6 mm diameter, Figure 2G). We imaged GCaMP-expressing neurons during 30 s bouts of OG-LED light pulses while the mice were freely behaving. To determine if the OG-LED light changes GCaMP emission, we measured changes in fluorescence from NAc neurons expressing GCaMP. We found that the OG-LED did not significantly change raw fluorescent counts during periods where the OG-LED was off vs. periods where the OG-LED was on (Interaction *F* = 3.68, pre vs. on, *p* = 0.99; post vs. on, *p* = 0.09, Figures 2H,I), suggesting that the OG-LED light does not cause significant changes in GCaMP emission. Importantly, we are not observing the significant increases in fluorescence that were observed in preparations without GCaMP (Figures 2A–F), suggesting that the noise associated with GCaMP imaging is far above the small increase in fluorescence from the OG-LED, as described above.

We next determined whether changes in temperature from the OG-LED would affect the stability of the EX-LED light output. To do this, we measured light output from the EX-LED during a 30 min recording session while delivering bouts of 30 s pulses of 10 mW/mm^2^ OG-LED light (Figure S1). We found that when we set the EX-LED to 1 mW/mm^2^, the EX-LED light output ranged from 0.995 to 1.007 mW/mm^2^ (0.7%, Figure S1). This measured stability of the EX-LED is critical for valid interpretations of OG-LED induced effects on Ca^2+^ dynamics. Collectively, these data demonstrate that the design of the integrated microscope prevents the OG-LED from interfering with Ca^2+^ imaging acquisition, analysis, or data interpretation.

### EX-LED crosstalk

This integrated microscope is designed for pairing GCaMP with red-shifted opsins (Figures 1A,B); thus we tested both excitatory and inhibitory red-shifted opsins to determine which red-shifted opsins are suitable for pairing with one-photon imaging of GCaMP. To that end, we quantified the extent to which the EX-LED activated red-shifted opsins in brain slices using whole-cell patch-clamp electrophysiology.

We first tested ChrimsonR, an excitatory red-shifted opsin (Klapoetke et al., 2014). For this, we performed whole-cell recordings from ChrimsonR-expressing neurons in brain slices during delivery of LEDs filtered with the OG-LED and EX-LED excitation filters (Figure S2A). We found that delivery of OG-LED light resulted in robust depolarization and optically-evoked spiking (Figures S2B–E). However, we also observed robust depolarization in response to delivery of EX-LED light (Figures S2B–F). In addition, at irradiances as low as 0.4 mW/mm^2^ we observed spiking in response to EX-LED illumination (Figure S2D). This data suggests that if one-photon Ca^2+^ imaging is combined with somatic ChrimsonR manipulation, the imaging light may depolarize the ChrimsonR-expressing cells *in vivo*.

**Figure 3:**
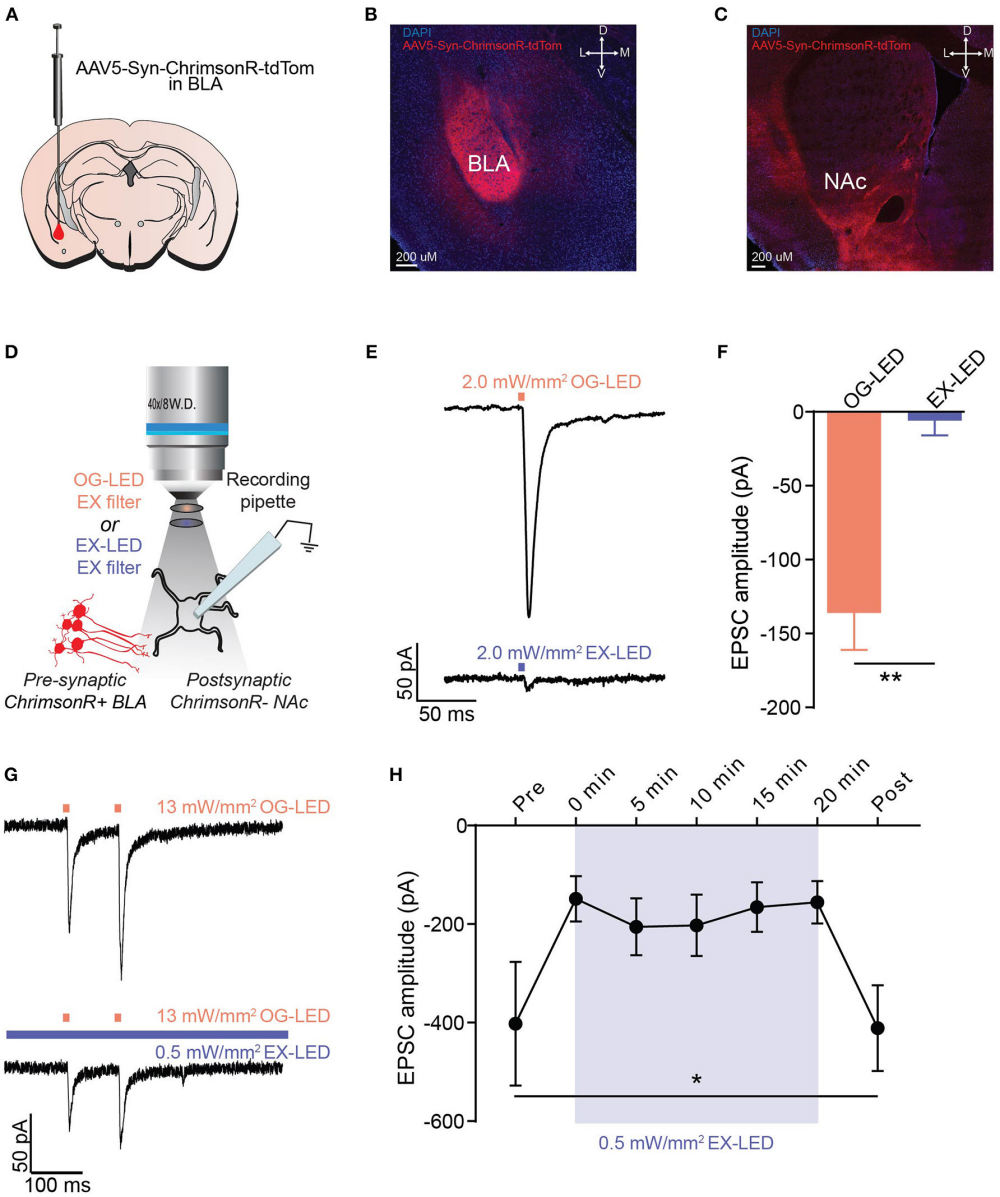
EX-LED has modest effects on pre-synaptic terminals expressing ChrimsonR. **(A)** Schematic of AAV5-Syn-ChrimsonR-tdTom injection into the BLA. **(B)** DAPI stained coronal section showing ChrimsonR-tdTom (red) expression in the BLA after virus injection. Scale bar = 200 μm. **(C)** DAPI stained coronal section showing ChrimsonR-tdTom (red) fiber expression in the NAc after virus injection in the BLA. No evidence of somatic expression of ChrimsonR in the NAc was observed. Scale bar = 200 μm. **(D)** Optically-evoked EPSCs were recorded from NAc neurons during optical stimulation of BLA-to-NAc fibers with LEDs filtered with the EX- and OG-LED excitation filters. **(E)** Representative traces showing current in a postsynaptic NAc neuron in response to 2 mW/mm^2^ of OG-LED stimulation and 2 mW/mm^2^ of EX-LED stimulation. **(F)** EPSC amplitude is significantly lower in response to EX-LED stimulation compared to OG-LED stimulation (*n* = 6 cells from *n* = 2 mice, paired *t*-test, *p* < 0.01). **(G)** Representative traces showing changes in current in a postsynaptic NAc neuron in response to 13 mW/mm^2^ pulsed OG-LED stimulation (top) and 13 mW/mm^2^ OG-LED pulsed stimulation during extended EX-LED stimulation (0.5 mW/mm^2^). **(H)** OG-LED light-evoked postsynaptic currents are significantly attenuated during exposure to simultaneous EX-LED (*n* = 6 cells from *n* = 5 mice, repeated-measures one-way ANOVA, *p* = 0.04. None of the Bonferroni multiple pairwise comparisons were significant). Error bar is SEM. *p < 0.05; **p < 0.01.

We next examined whether one-photon imaging can be combined with photostimulation of terminals expressing ChrimsonR. Since the integrated microscope enables optogenetics and Ca^2+^ imaging within the same field of view, one application of this technology is imaging somatic Ca^2+^ activity while simultaneously stimulating opsin-expressing terminals arising from a distal brain region. For this test, we recorded optically-evoked excitatory post synaptic currents (EPSCs) from postsynaptic neurons in the NAc while optically stimulating terminals expressing ChrimsonR from the BLA (Figures 3A–D). We found that delivering OG-LED light resulted in large optically-evoked EPSCs in postsynaptic neurons (Figures 3E,F). However, we found that exposure to EX-LED light resulted in minimal optically-evoked EPSCs in postsynaptic neurons, even at 2 mW/mm^2^, the maximum EX-LED irradiance the system allows for imaging (*t* = 5.162, *p* < 0.01, Figures 3E,F). During typical imaging sessions, the EX-LED is on for the entire imaging session, at a relatively low irradiance, while the OG-LED is pulsed at a high irradiance. To investigate how these *in vivo* light parameters affect the cellular response, we next measured OG-LED optically-evoked EPSCs before, during, and after extended EX-LED exposure (Figures 3G,H). Interestingly, we found that concurrent exposure to the EX-LED attenuates the OG-LED-induced optically-evoked EPSC (*F* = 5.414, *p* = 0.04, Figures 3G,H). This attenuation was immediate, and reversible (Figure 3H). In a control set of cells, where the neurons received OG-LED stimulation but no EX-LED stimulation, we observed no significant change in light-evoked current over the 20 min of recording (repeated-measures one-way ANOVA, *F* = 0.45, *p* = 0.63).

We next tested two red-shifted inhibitory opsins, NpHR3.0 and Jaws (Gradinaru et al., 2010; Chuong et al., 2014). For this, we performed whole-cell recordings from NpHR3.0- or Jaws-expressing neurons in brain slices during delivery of LEDs filtered with the OG-LED and EX-LED excitation filters (Figures S3, S4). Both NpHR3.0- and Jaws-expressing neurons showed increasing amounts of hyperpolarization in response to increasing OG-LED irradiances (Figures S3, S4). While we did observe some amount of hyperpolarization in response to the EX-LED, the amount of crosstalk was much less than with EX-LED activation of somatic ChrimsonR (Figures S3, S4). In addition, the crosstalk is minimal at the lower irradiances (<1 mW/mm^2^) that are typically used for *in vivo* one-photon Ca^2+^ imaging (Ziv et al., 2013; Betley et al., 2015; Jennings et al., 2015).

These data suggest that this integrated microscope can be used for pairing one-photon Ca^2+^ imaging of GCaMP with one-photon full-field somatic and terminal inhibition using NpHR or Jaws, or terminal excitation using ChrimsonR.

### Thermal characterization of OG-LED-induced heating of brain tissue

We designed the OG-LED to perform at a maximum irradiance of 10 mW/mm^2^ at a 100% duty cycle for inhibitory optogenetic experiments, or 35 mW/mm^2^ at a 25% duty cycle for excitatory optogenetic experiments where the LED is pulsed. We quantified the degree to which varying OG-LED irradiances increases the temperature of brain tissue at the imaging plane. In brain slices, the OG-LED increased the temperature at the imaging plane (200 μm below the bottom of the 4 × 1 mm GRIN lens) <1°C, even at the maximum duty cycle and irradiance (Figure S5). Actual temperature changes *in vivo*, however, will likely be <1°C due to heat dissipation through blood flow.

### Simultaneous Ca^2+^ imaging and optogenetic manipulation in a freely-behaving mouse

To demonstrate the feasibility of simultaneous full-field optogenetic manipulation and cellular-resolution Ca^2+^ imaging in freely-behaving mice using this integrated technology, we investigated the BLA-to-NAc circuit. The connection between the BLA and NAc plays a critical role in reward circuitry underlying emotion, reward, and motivated behaviors (Setlow et al., 2002; Ambroggi et al., 2008; Stuber et al., 2011; Janak and Tye, 2015). However, how information transmission within the BLA-to-NAc circuit translates to reward remains unclear. Here, we use this integrated technology to explore how selective activation of the BLA-to-NAc circuit is causally linked to NAc neural circuit dynamics that encode reward behavior.

**Figure 4:**
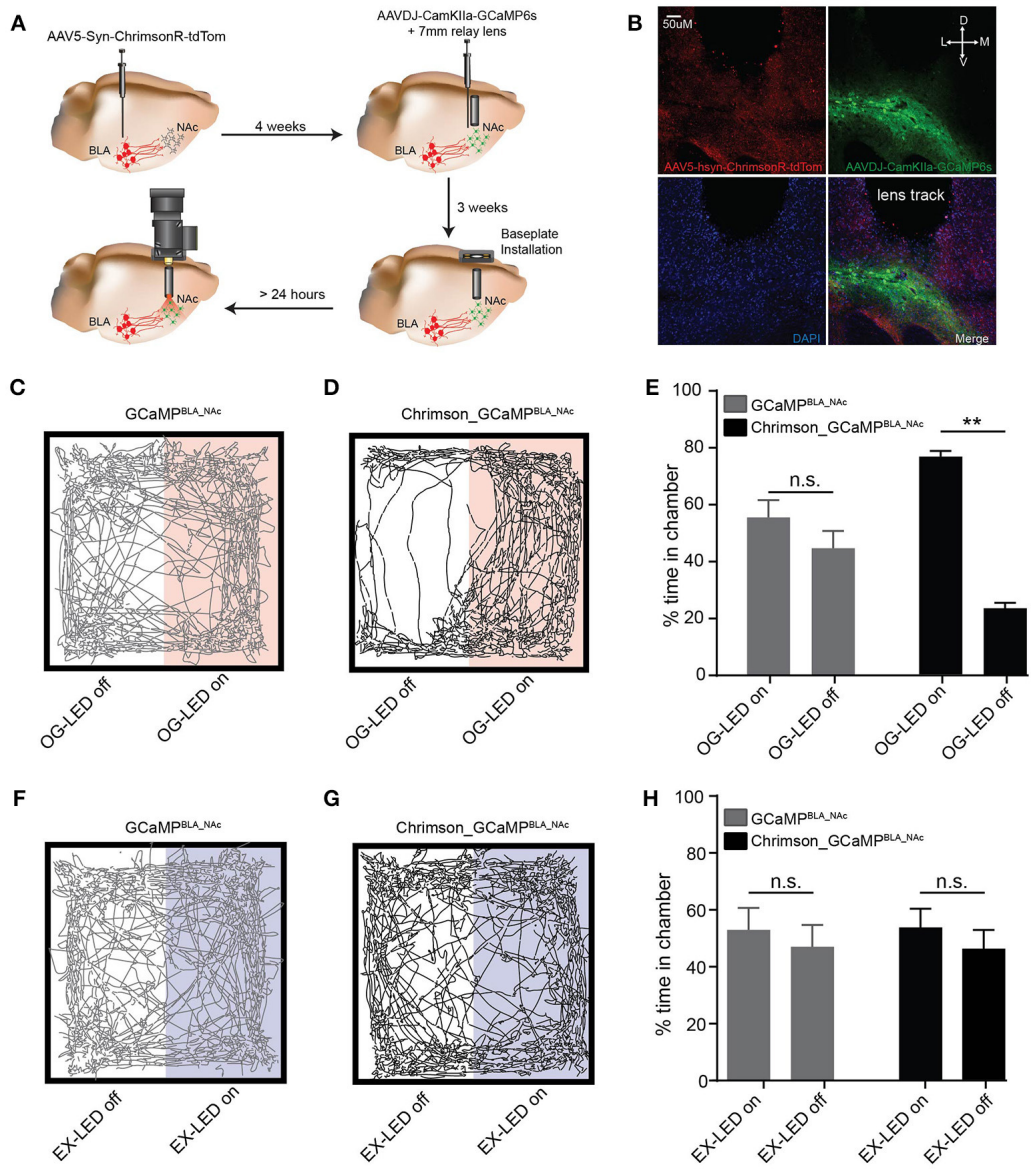
*In vivo* activation of BLA-to-NAc circuit using an integrated microscope produces a rewarding phenotype. **(A)** Schematic of surgical workflow for *in vivo* simultaneous imaging and optogenetic experiments. **(B)** DAPI stained coronal section showing ChrimsonR-tdTom (red) fiber expression in the NAc after virus injection in the BLA (top left panel), GCaMP (green) somatic expression in the NAc after virus injection in the NAc (top right panel), and lens track from a 7 mm (0.6 mm diameter) implanted GRIN lens above the NAc (bottom left and right panels). Scale bar = 50 μm. **(C,D)** Example OG-LED real-time place preference activity plots from a representative GCaMP^BLA_NAc^ mouse **(C)** and a representative Chrimson_GCaMP^BLA_NAc^ mouse **(D)**. **(E)** GCaMP^BLA_NAc^ mice spend equal time in the OG-LED on and OG-LED off chambers (*n* = 7 mice, paired *t*-test, *p* = 0.30) Chrimson_GCaMP^BLA_NAc^ mice spend significantly more time in the OG-LED on chamber (*n* = 7 mice, paired *t*-test, *p* < 0.01). **(F,G)** Example EX-LED real-time place preference activity plots from a representative GCaMP^BLA_NAc^ mouse **(F)** and a representative Chrimson_GCaMP^BLA_NAc^ mouse **(G)**. **(H)** GCaMP^BLA_NAc^ mice and Chrimson_GCaMP^BLA_NAc^ spend equal time in the EX-LED on and EX-LED off chambers (GCaMP^BLA_NAc^: *n* = 7 mice, paired *t*-test, *p* = 0.71; Chrimson_GCaMP^BLA_NAc^: *n* = 8 mice, paired *t*-test, *p* = 0.60). All error bars are SEM. **p < 0.01.

To enable simultaneous optogenetic manipulation and Ca^2+^ imaging in the same field of view, we first injected AAV5-ChrimsonR-tdTomato in the BLA (Figure 4A). Four weeks later we injected AAVDJ-CamKIIa-GCaMP6s in the NAc and inserted a 7 mm GRIN lens (0.6 mm diameter) above the NAc, allowing for simultaneous BLA terminal stimulation paired with Ca^2+^ imaging of NAc neurons (Chrimson_GCaMP^BLA_NAc^ mice; Figures 4A,B). A control group of mice received the same surgical protocol, except no ChrimsonR was injected into the BLA (GCaMP^BLA_NAc^). Two to four weeks later, we installed a docking station for the integrated microscope in both groups of mice. This allows for the microscope to be removed from the mouse between recordings sessions. Further, this docking station allows for the ability to return to the same focal plane, enabling longitudinal imaging of the same neuronal population (Ziv et al., 2013). Prior to the imaging and optogenetic sessions, mice were habituated to the microscope and to the behavioral box. During the habituation session, we found no significant difference in time spent in left vs. right side of the chamber, velocity, or distance traveled between the Chrimson_GCaMP^BLA_NAc^ and GCaMP^BLA_NAc^ groups (% time on left vs. right side of chamber: GCaMP^BLA_NAc^ mice *t* = 0.34, *p* = 0.75, Chrimson_GCaMP^BLA_NAc^ *t* = 1.12, *p* = 0.30; velocity: *t* = 0.21, *p* = 0.84, distance traveled: *t* = 0.13, *p* = 0.90 Figure S6).

We first sought to demonstrate that the OG-LED was able to deliver an irradiance high enough to activate red-shifted opsins to promote the intended behavioral response. Optogenetic activation of BLA terminals in the NAc has previously been demonstrated to induce a reinforcing and rewarding phenotype (Stuber et al., 2011; Britt et al., 2012). Using this integrated microscope, we were able to induce a significant real-time place preference to a side of a chamber that was paired with OG-LED stimulation trains in Chrimson_GCaMP^BLA_NAc^ mice (*t* = 13.8, *p* < 0.01, Figures 4C–E). We found that GCaMP^BLA_NAc^ mice did not show a place preference (*t* = 1.149, *p* = 0.30, Figures 4C–E), indicating that the presence of OG-LED light alone in the absence of opsin expression is not sufficient to induce a rewarding phenotype. We next investigated whether a real-time place preference could be evoked with the EX-LED light alone. If the EX-LED is activating ChrimsonR to a significant degree, we would expect the Chrimson_GCaMP^BLA_NAc^ mice to show a preference for a side of the chamber paired with EX-LED light. We found that a real-time place preference experiment, in which one side of the chamber was paired with EX-LED stimulations did not result in a significant preference in the Chrimson_GCaMP^BLA_NAc^ or GCaMP^BLA_NAc^ groups (Chrimson_GCaMP^BLA_NAc^: *t* = 0.56, *p* = 0.60; GCaMP^BLA_NAc^: *t* = 0.39, *p* = 0.71; Figures 4F–H). This EX-LED control data, combined with the patch-clamp electrophysiology data (Figure 3), suggests that activation of ChrimsonR terminals with EX-LED light is not likely to significantly alter the outcome of the optogenetic experiments.

**Figure 5:**
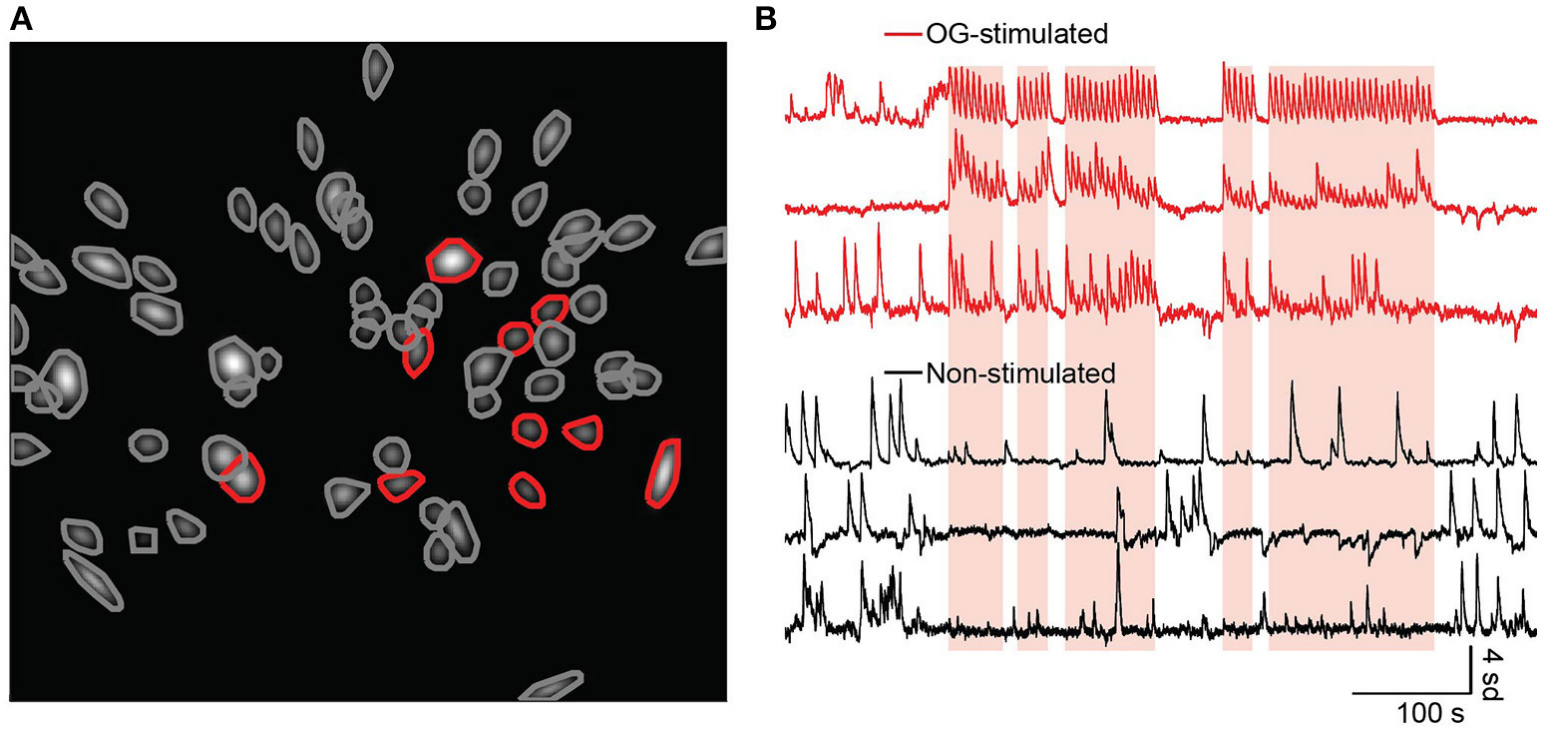
A subset of NAc neurons show an increase in Ca^2+^ activity following optogenetic activation of the BLA-to-NAc circuit. **(A)** ROIs fit with 2D Gaussians identified from representative Chrimson_GCaMP^BLA_NAc^ mouse during a RTPP session, along with their contours. Cells with red contours were found to be OG-stimulated, gray contours are non-stimulated. **(B)** Example traces from OG-stimulated (red) and non-stimulated (black) cells. Times when the mouse was in the OG-LED on chamber are indicated with overlaid orange translucent rectangles.

Lastly, we investigated whether NAc neurons are modulated during optogenetic stimulation of BLA terminals during this real-time place preference task. After data collection we processed the imaging movies, identified cells, and fit linear encoders to determine which cells were modulated by the OG-LED stimulation. Most cells exhibited Ca^2+^ events which were within the normal range for GCaMP6s (Chen et al., 2013, see Figure S7 for summary histograms). Cells were excluded from the analysis if they exhibited a Ca^2+^ event rate of <0.01 Hz, or a Ca^2+^ event decay time of <300 ms. For the GCaMP^BLA_NAc^ mice, an average of 40 ± 20 cells were detected (mean ± SD, *n* = 5, two GCaMP^BLA_NAc^ mice excluded due to low yield), while in Chrimson_GCaMP^BLA_NAc^ mice we detected on average 50 ± 20 cells (mean ± SD, *n* = 5). We reliably detected a subset of NAc cells whose event rates were modulated by the OG-LED stimulation in the Chrimson_GCaMP^BLA_NAc^ mice (Figures 5A,B, Movie S1). For the Chrimson_GCaMP^BLA_NAc^ mice, an average of 21 ± 11% of the cells were OG-stimulated (mean ± SD, *n* = 5).

## Discussion

Here we have developed and optimized an integrated light-weight microscope that allows for one-photon cellular-resolution Ca^2+^ imaging paired with optogenetic manipulation within the same field of view in freely-behaving mice. We have verified the utility of this technology by investigating the causal link between the BLA-to-NAc circuit, NAc Ca^2+^ dynamics, and reward-related behavior. This integrated microscope can likely support a wide range of applications, including Ca^2+^ imaging paired with terminal stimulation, and Ca^2+^ imaging paired with somatic or terminal inhibition. For example, one could investigate the causal relationship of a particular input into a network on behavior and network coding, by inhibiting presynaptic terminals expressing inhibitory opsins during a behavior that has been shown to induce a particular encoding pattern.

Although our data suggests that this integrated microscope can likely support the applications described above, there are a number of caveats and control experiments to consider. While our experiments demonstrated minimal crosstalk with inhibitory opsins and terminal excitatory opsins, crosstalk is not completely eliminated. Thus, it is critical that proper controls are conducted to determine if this small amount of crosstalk during imaging sessions could impact interpretation of the data. These control experiments can include a combination of investigating the effect of the EX-LED on the electrical activity of networks expressing opsins in brain slices (Figure 3), assessing the effect of the EX-LED on behavior (Figures 4F–H), and measuring the effect of the OG-LED on GCaMP activity in mice that express no opsin (Figures 2G–I).

It should also be noted that the majority of our biological crosstalk data (the effect of imaging light on opsins) was obtained in brain slices, rather than *in vivo*. Because of the technical challenges associated with electrophysiological recordings paired with imaging *in vivo*, we found that measuring biological crosstalk in brain slices was a more straightforward approach. However, it is likely that our biological crosstalk measurements are an overestimate of what we would observe *in vivo*. *In vivo*, the imaging plane of the GRIN lens is typically 200 microns below the bottom of the lens. Thus, the irradiance of the EX-LED at the imaging plane is less than what we used in brain slices, since light scatters in brain tissue. Nonetheless, it is critical to conduct appropriate control experiments for measuring the effect of the EX-LED on opsins, as described above.

We also observed in brain slices, that the imaging light attenuates the effect of the optogenetic light on postsynaptic neuronal responses during ChrimsonR presynaptic terminal stimulation (Figures 3G,H). While this effect has not previously been reported for ChrimsonR, a similar effect has been observed for Channelrhodopsin-2 (ChR2). ChR2, whose activation peaks at 480 nm, has a red-shifted (520 nm) intermediate which results in an inactivated ChR2 protein (Bamann et al., 2008; Verhoefen et al., 2010). Thus, it is possible that we are observing an inactivation of ChrimsonR, due to excitation of a blue-light absorbing intermediate. Alternatively, it is possible that excitation could be transferred from the excited tagged fluorescent protein (tdTomato) to a ChrimsonR photocycle intermediate, interrupting the photocycle and consequently preventing channel opening. Both possibilities are yet to be tested. Nonetheless, although we observed attenuation from the EX-LED, our *in vivo* data suggests that the OG-LED irradiance that we used is sufficient to cause the intended behavioral (Figure 4) and cellular (Figure 5) effects, even in the presence of the EX-LED. While we only measured the effects of extended EX-LED on OG-LED responses when ChrimsonR is expressed presynaptically, it is also possible that the EX-LED could result in a similar attenuation to circuits expressing inhibitory opsins, such as NpHR or Jaws. Thus, it is important for future experiments requiring the use of inhibitory opsins, to conduct similar experiments as shown in Figures 3G,H.

Several imaging and optogenetic parameters need to be set to conduct these integrated experiments. This includes the EX-LED irradiance, exposure time, gain, OG-LED irradiance, and OG-LED pulse protocol. To minimize GCaMP photobleaching, and opsin crosstalk, the EX-LED should be set to a low irradiance, but high enough to elicit the appropriate signal-to-noise ratio (SNR) required for the experiment. Increasing the sensor gain and exposure time can aid in signal detection, and thus be used in combination with a lower EX-LED irradiance to acquire the appropriate SNR. These calibration experiments can be conducted prior to the experiment to optimize the imaging parameters. In our hands, we found that between 5 and 35 mW/mm^2^ of OG-LED irradiance was sufficient to induce the intended cellular effect (increase calcium activity during OG-LED pulse trains) in the BLA-to-NAc circuit. This variance likely arose from differences in ChrimsonR presynaptic terminal expression. To determine the appropriate light levels to use, we conducted calibration procedures prior to the experiments, in which we slowly ramped up the OG-LED light levels until we observed a robust effect on GCaMP activity, visualized under df/f in real time.

Finally, we have demonstrated that Ca^2+^ imaging paired with one-photon somatic activation remains a biological challenge since all published red-shifted excitatory opsins are activated by blue light (Chow et al., 2010; Gradinaru et al., 2010; Yizhar et al., 2011b; Lin et al., 2013; Chuong et al., 2014; Klapoetke et al., 2014). Further engineering of red-shifted opsins to narrow and/or further red-shift their action spectra could enable routine simultaneous somatic stimulation with one-photon Ca^2+^ imaging. Alternatively, advancements in red-shifted Ca^2+^ indicators could enable one-photon calcium imaging paired with blue-shifted opsins, such as ChR2, which show negligible absorbance above a particular threshold wavelength (Nagel et al., 2003; Zhang et al., 2007), a method that has already been demonstrated with two-photon imaging (Forli et al., 2018). Future microscopes based on the technology presented here would be well suited for this alternate-color approach to simultaneous optogenetic manipulation paired with calcium imaging.

One exciting potential advancement of this technology is the possibility of closed loop optogenetic manipulation that is gated by pre-defined Ca^2+^ responses. Advances in real-time data analysis, including faster motion-correction algorithms, offer an exciting avenue for this. The potential for an all optical closed loop platform would allow for the testing of sophisticated causal hypotheses that link circuit dynamics with emergent behavior, system states, and plasticity. These experiments would likely have great utility for translational approaches seeking to use a real-time biofeedback approach to neural modulation.

Since one-photon Ca^2+^ imaging is limited by the inability to image sub-cellular structures, integration of one-photon and two-photon microscopy could allow for the benefits of one- and two-photon imaging techniques to be combined in a single experiment. Advancements in hardware and registration algorithms could enable seamless transition between one- and two-photon experiments in the same mouse, enabling sub-cellular imaging with a head-fixed mouse using two-photon, and cellular dynamics imaging during natural behavior with a freely-behaving mouse using one-photon. In addition, registration between one-photon and two-photon could elucidate the differences in physiological relevance between manipulating activity-dependent defined neurons (with two-photon experiments) vs. full field stimulation of opsin-expressing neurons or terminals (with one-photon).

Typical Ca^2+^ imaging experiments using viral strategies to express genetically encoded Ca^2+^ indicators often result in a population that is targeted by a single-gene, or a single projection. Co-registering imaged neurons *in vivo* with histology can allow experimenters to *post-hoc* classify their recorded neurons with a higher degree of categorization. Co-registering imaged neurons with fluorescence *in situ* hybridization can enable the experimenter to assign a rich molecular profile to recorded neurons. Alternatively, co-registering imaged neurons with tissue clearing techniques, such as CLARITY, can allow the experimenter to identify the recorded neurons based on a brain-wide projection target (Chung et al., 2013).

Investigating the causal relationship between neural circuit dynamics, brain function, and behavior is a critical step to understanding how the brain works, both in health and disease. While Ca^2+^ imaging and optogenetics alone have contributed greatly toward our understanding of neural circuits, the combination of these two techniques has the promise to greatly expand the types of questions neuroscientists are able to address. The integration of simultaneous neural circuit monitoring and manipulation will allow investigators to test sophisticated causal hypotheses that link neural circuit dynamics with behavior, system states, and plasticity.

## Datasets are available on request

The raw data supporting the conclusions of this manuscript will be made available by the authors, without undue reservation, to any qualified researcher.

## Ethics statement

All procedures were conducted in accordance with the Guide for the Care and Use of Laboratory Animals, as adopted by the National Institutes for Health, and with approval of the Institutional Animal Care and Use Committee.

## Author contributions

Optical components were designed and developed by MT. Mechanical components were designed and developed by SM. Optical spectrometry and optical crosstalk was performed by MT. Thermal testing was conducted by AT. Image stability testing was performed by CF. All biological testing was conducted by AS, KZ, and SG. Ca^2+^ data analysis was conducted by MS. The manuscript was written by AS with comments from all authors. SO supervised the project.

### Conflict of interest statement

The authors report competing financial interests: AS, MS, SG, KZ, SM, AT, CF, MT, and SO are paid employees at Inscopix.

## Supplementary material

Movie S1 Representative synchronized *in vivo* Ca^2+^, optogenetic, and behavior movie demonstrating OG-modulated Ca^2+^ activity in NAc neurons during BLA-to-NAc activation. DF/F Ca^2+^ movie synched with behavioral video from the RTPP task. NAc neurons display enhanced Ca^2+^ activity during optogenetic activation.

Figure S1 EX-LED is stable during OG-LED light pulses. *Ex-vivo* EX-LED measurements of light output in response to 30 s pulses of 10 mW/mm^2^ OG-LED (100% duty cycle). Measured EX-LED output ranged from 0.995 to 1.007 mW/mm^2^ when set to 1 mW/mm^2^.

Figure S2 EX-LED induces robust depolarization of somas expressing ChrimsonR at typical imaging irradiances. **(A)** AAV5-Syn-ChrimsonR-tdTom was injected into the somatosensory cortex. Changes in depolarization were measured from cortical neurons expressing ChrimsonR in response to LEDs filtered with the EX- and OG-LED excitation filters. **(B)** Representative traces showing current in a neuron expressing ChrimsonR in response to 0.4 mW/mm^2^ of OG-LED stimulation and 0.4 mW/mm^2^ of EX-LED stimulation. **(C)** Quantification of changes in current (pA) during OG-LED and EX-LED light pulses. (Data points, mean ± SEM; *n* = 11 neurons). **(D)** Representative traces showing voltage in a neuron expressing ChrimsonR in response to 0.4 mW/mm^2^ of OG-LED stimulation and 0.4 mW/mm^2^ of EX-LED stimulation. **(E)** Quantification of number of spikes during OG-LED and EX-LED light pulses. (Data points, mean ± SEM; *n* = 8 neurons). **(F)** Quantification of number of spikes as a percentage of OG-LED (Spikes = (EX-LED Spikes/OG-LED Spikes)^*^100; Data points, mean ± SEM; *n* = 8 neurons). All error bars are SEM.

Figure S3 EX-LED induces minimal hyperpolarization of somas expressing NpHR3.0 at typical imaging irradiances. **(A)** AAV5-CamKIIa-NpHR3.0-mCherry was injected into the somatosensory cortex. Changes in hyperpolarization were measured from cortical neurons expressing NpHR3.0 in response to LEDs filtered with the EX- and OG-LED excitation filters. **(B)** Representative traces showing current in a neuron expressing NpHR3.0 in response 1.0 mW/mm^2^ of OG-LED stimulation and 1.0 mW/mm^2^ of EX-LED stimulation. **(C)** Quantification of changes in current (pA) during OG-LED and EX-LED light pulses. (Data points, mean ± SEM; *n* = 9 neurons). **(D)** Representative traces showing voltage in a neuron expressing NpHR3.0 in response to a 200 pA current step (baseline) followed by a 200 pA current step combined with 1.0 mW/mm^2^ of OG-LED stimulation (left) and 1.0 mW/mm^2^ of EX-LED stimulation (right). **(E)** Quantification of % inhibition during OG-LED and EX-LED light pulses [% Inhibition = ((baseline spikes – LED spikes)/baseline spikes × 100) Data points, mean ± SEM; *n* = 7 neurons]. All error bars are SEM.

Figure S4 EX-LED induces minimal hyperpolarization of somas expressing Jaws at typical imaging irradiances. **(A)** AAV5-CamKIIa-Jaws-KCG-GFP-ER2 was injected into the somatosensory cortex. Changes in hyperpolarization were measured from cortical neurons expressing Jaws in response to LEDs filtered with the EX- and OG-LED excitation filters. **(B)** Representative traces showing current in a neuron expressing Jaws in response 1.0 mW/mm^2^ of OG-LED stimulation and 1.0 mW/mm^2^ of EX-LED stimulation. **(C)** Quantification of changes in current (pA) during OG-LED and EX-LED light pulses. (Data points, mean ± SEM; *n* = 7 neurons). **(D)** Representative traces showing voltage in a neuron expressing Jaws in response to a 200 pA current step (baseline) followed by a 200 pA current step combined with 1.0 mW/mm^2^ of OG-LED stimulation (left) and 1.0 mW/mm^2^ of EX-LED stimulation (right). **(E)** Quantification of % inhibition during OG-LED and EX-LED light pulses [% Inhibition = ((baseline spikes – LED spikes)/baseline spikes × 100) Data points, mean ± SEM; *n* = 7 neurons]. All error bars are SEM.

Figure S5 Various OG-LED optogenetic pulse parameters change the temperature of brain slices <1°C. **(A)** Representative traces of temperature vs. time. **(B)** Quantification of temperature change (average temperature over last 100 s of OG-LED – average temperature during 30 s baseline, Data points, mean ± SEM from *n* = 2 technical replicates and *n* = 2 biological replicates).

Figure S6 GCaMP^BLA_NAc^ mice and Chrimson_GCaMP^BLA_NAc^ display similar behavior during habituation. **(A)** GCaMP^BLA_NAc^ mice and Chrimson_GCaMP^BLA_NAc^ spend equal times on the left and right side of the behavioral chamber (GCaMP^BLA_NAc^ mice:, *n* = 7 mice, paired *t*-test, *p* = 0.75; Chrimson_GCaMP^BLA_NAc^ mice: *n* = 8 mice, paired *t-*test, *p* = 0.30). **(B)** Velocity was not significantly different in GCaMP^BLA_NAc^ mice and Chrimson_GCaMP^BLA_NAc^ during the 30 min habituation session (*n* = 7 GCaMP^BLA_NAc^ mice; *n* = 8 Chrimson_GCaMP^BLA_NAc^ mice; unpaired *t*-test, *p* = 0.84). **(C)** Distance traveled was not significantly different in GCaMP^BLA_NAc^ mice and Chrimson_GCaMP^BLA_NAc^ during the 30 min habituation session (*n* = 7 GCaMP^BLA_NAc^ mice; *n* = 8 Chrimson_GCaMP^BLA_NAc^ mice, unpaired *t*-test, *p* = 0.90). All error bars are SEM.

Figure S7 Statistics from cells included in linear filter encoder. **(A)** Histogram of average Ca^2+^ event rise times across all cells (*n* = 463 cells total from *n* = 5 GCaMP^BLA_NAc^ mice and *n* = 5 Chrimson_GCaMP^BLA_NAc^ mice). **(B)** Histogram of average Ca^2+^ event decay times across all cells (*n* = 463). Cells were excluded if they had an event rate of <0.01 Hz, or a decay time of <300 ms. **(C)** A histogram of event rates across all cells. **(D)** Linear filter encoder *R*^2^ values for all cells.
