# Supplementary figures and images for: Simultaneous Optogenetics and Cellular Resolution Calcium Imaging During Active Behavior Using a Miniaturized Microscope
Source: Front Neurosci. 2018 Jul 24;12:496. doi: 10.3389/fnins.2018.00496 (PMC6066578; doi:10.3389/fnins.2018.00496)

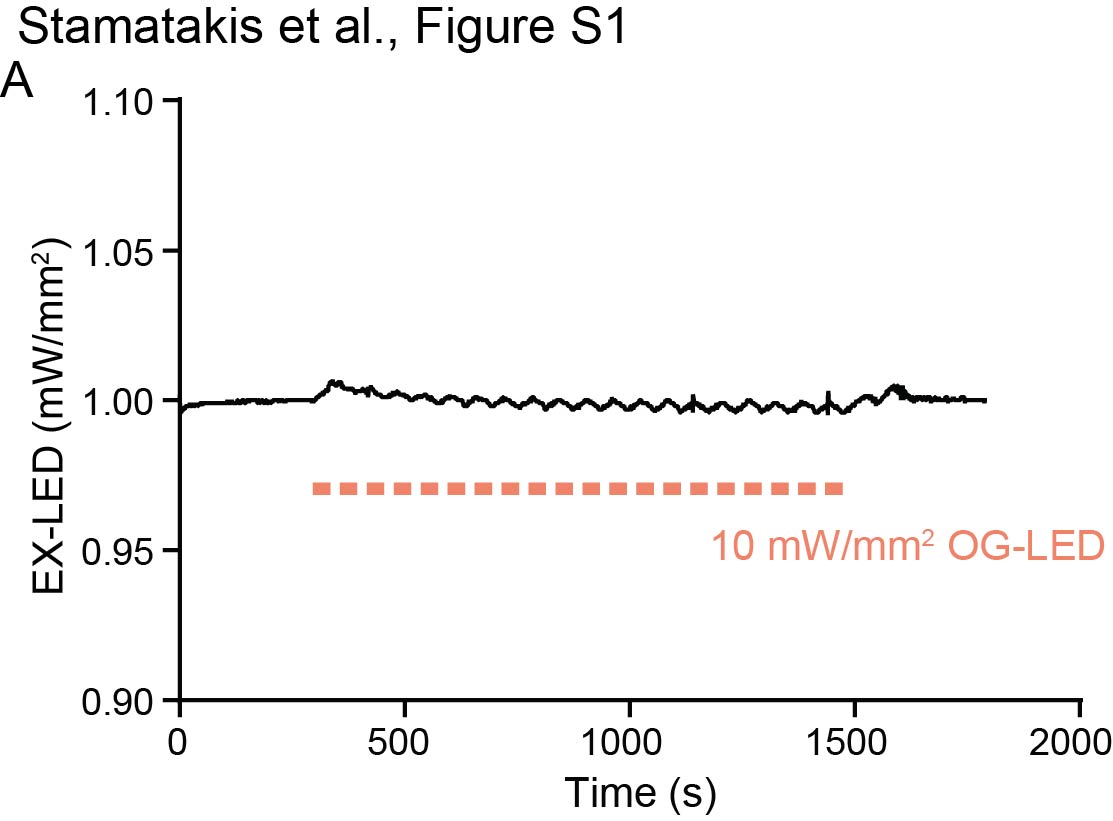

Supplement: Figure S1 — EX-LED is stable during OG-LED light pulses. Ex-vivo EX-LED measurements of light output in response to 30 s pulses of 10 mW/mm2 OG-LED (100% duty cycle). Measured EX-LED output ranged from 0.995 to 1.007 mW/mm2 when set to 1 mW/mm2. [file Image_1.JPEG]

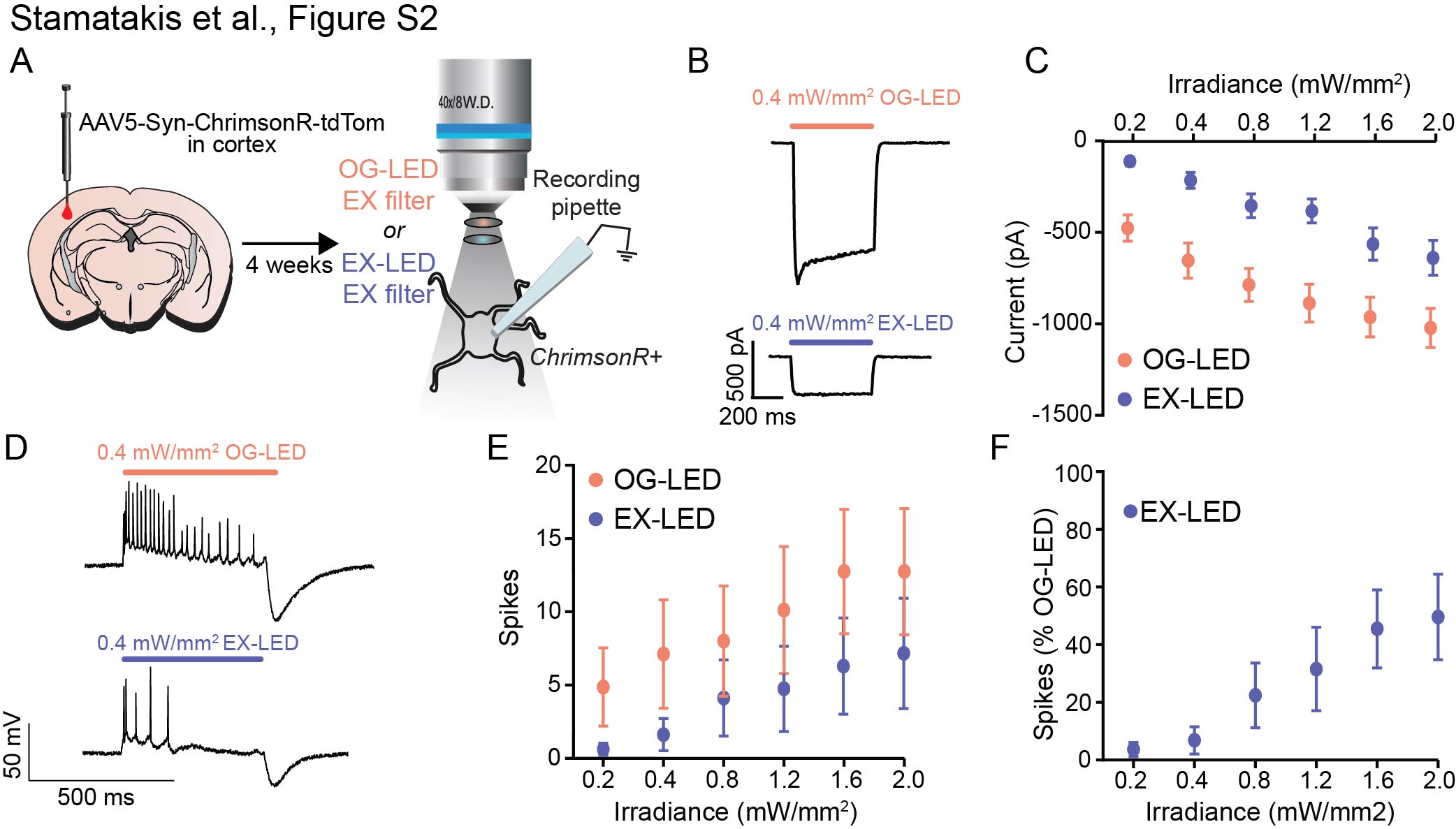

Supplement: Figure S2 — EX-LED induces robust depolarization of somas expressing ChrimsonR at typical imaging irradiances. (A) AAV5-Syn-ChrimsonR-tdTom was injected into the somatosensory cortex. Changes in depolarization were measured from cortical neurons expressing ChrimsonR in response to LEDs filtered with the EX- and OG-LED excitation filters. (B) Representative traces showing current in a neuron expressing ChrimsonR in response to 0.4 mW/mm2 of OG-LED stimulation and 0.4 mW/mm2 of EX-LED stimulation. (C) Quantification of changes in current (pA) during OG-LED and EX-LED light pulses. (Data points, mean ± SEM; n = 11 neurons). (D) Representative traces showing voltage in a neuron expressing ChrimsonR in response to 0.4 mW/mm2 of OG-LED stimulation and 0.4 mW/mm2 of EX-LED stimulation. (E) Quantification of number of spikes during OG-LED and EX-LED light pulses. (Data points, mean ± SEM; n = 8 neurons). (F) Quantification of number of spikes as a percentage of OG-LED (Spikes = (EX-LED Spikes/OG-LED Spikes)*100; Data points, mean ± SEM; n = 8 neurons). All error bars are SEM. [file Image_2.JPEG]

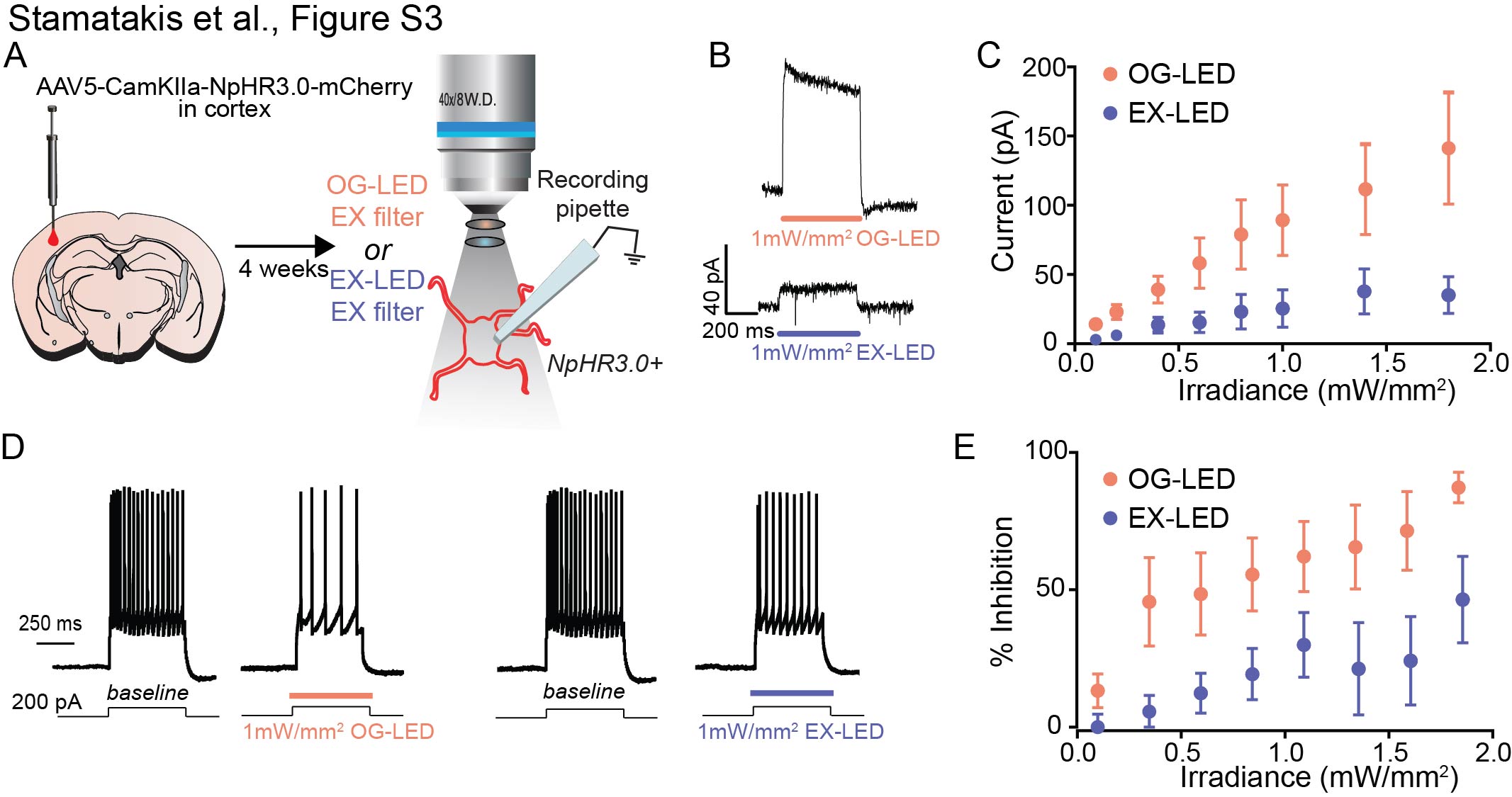

Supplement: Figure S3 — EX-LED induces minimal hyperpolarization of somas expressing NpHR3.0 at typical imaging irradiances. (A) AAV5-CamKIIa-NpHR3.0-mCherry was injected into the somatosensory cortex. Changes in hyperpolarization were measured from cortical neurons expressing NpHR3.0 in response to LEDs filtered with the EX- and OG-LED excitation filters. (B) Representative traces showing current in a neuron expressing NpHR3.0 in response 1.0 mW/mm2 of OG-LED stimulation and 1.0 mW/mm2 of EX-LED stimulation. (C) Quantification of changes in current (pA) during OG-LED and EX-LED light pulses. (Data points, mean ± SEM; n = 9 neurons). (D) Representative traces showing voltage in a neuron expressing NpHR3.0 in response to a 200 pA current step (baseline) followed by a 200 pA current step combined with 1.0 mW/mm2 of OG-LED stimulation (left) and 1.0 mW/mm2 of EX-LED stimulation (right). (E) Quantification of % inhibition during OG-LED and EX-LED light pulses [% Inhibition = ((baseline spikes – LED spikes)/baseline spikes × 100) Data points, mean ± SEM; n = 7 neurons]. All error bars are SEM. [file Image_3.JPEG]

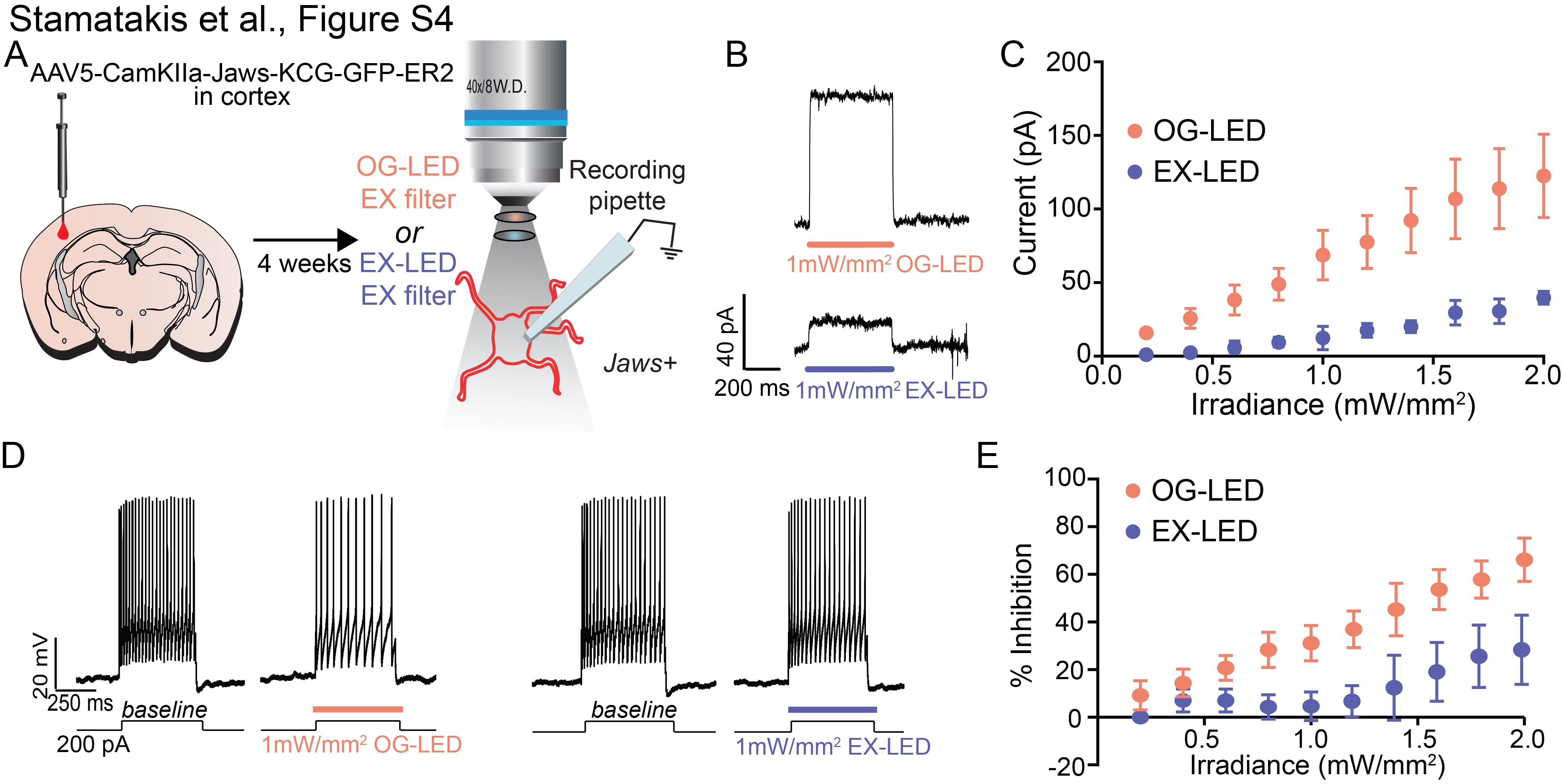

Supplement: Figure S4 — EX-LED induces minimal hyperpolarization of somas expressing Jaws at typical imaging irradiances. (A) AAV5-CamKIIa-Jaws-KCG-GFP-ER2 was injected into the somatosensory cortex. Changes in hyperpolarization were measured from cortical neurons expressing Jaws in response to LEDs filtered with the EX- and OG-LED excitation filters. (B) Representative traces showing current in a neuron expressing Jaws in response 1.0 mW/mm2 of OG-LED stimulation and 1.0 mW/mm2 of EX-LED stimulation. (C) Quantification of changes in current (pA) during OG-LED and EX-LED light pulses. (Data points, mean ± SEM; n = 7 neurons). (D) Representative traces showing voltage in a neuron expressing Jaws in response to a 200 pA current step (baseline) followed by a 200 pA current step combined with 1.0 mW/mm2 of OG-LED stimulation (left) and 1.0 mW/mm2 of EX-LED stimulation (right). (E) Quantification of % inhibition during OG-LED and EX-LED light pulses [% Inhibition = ((baseline spikes – LED spikes)/baseline spikes × 100) Data points, mean ± SEM; n = 7 neurons]. All error bars are SEM. [file Image_4.PNG]

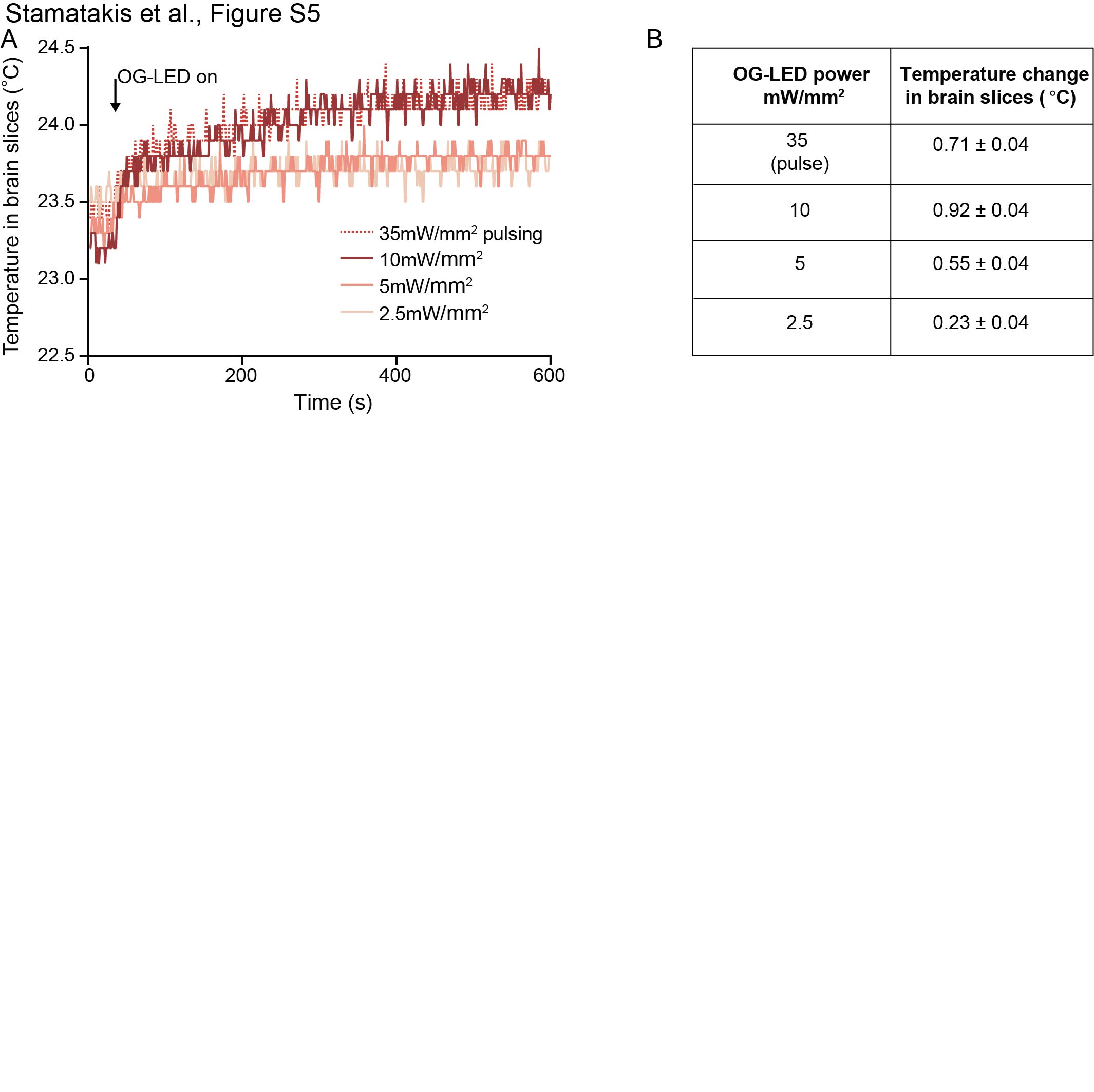

Supplement: Figure S5 — Various OG-LED optogenetic pulse parameters change the temperature of brain slices <1°C. (A) Representative traces of temperature vs. time. (B) Quantification of temperature change (average temperature over last 100 s of OG-LED – average temperature during 30 s baseline, Data points, mean ± SEM from n = 2 technical replicates and n = 2 biological replicates). [file Image_5.JPEG]

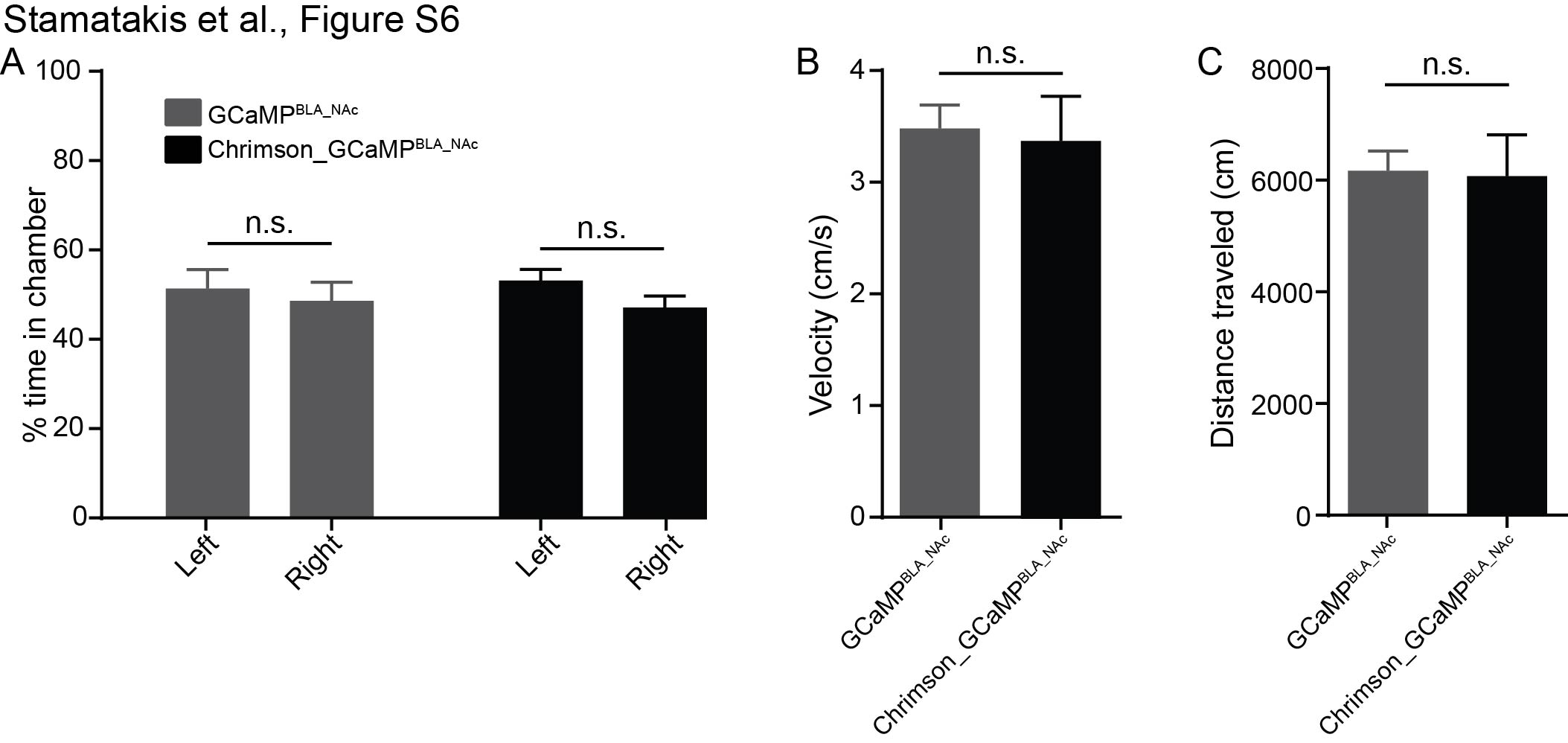

Supplement: Figure S6 — GCaMPBLA_NAc mice and Chrimson_GCaMPBLA_NAc display similar behavior during habituation. (A) GCaMPBLA_NAc mice and Chrimson_GCaMPBLA_NAc spend equal times on the left and right side of the behavioral chamber (GCaMPBLA_NAc mice:, n = 7 mice, paired t-test, p = 0.75; Chrimson_GCaMPBLA_NAc mice: n = 8 mice, paired t-test, p = 0.30). (B) Velocity was not significantly different in GCaMPBLA_NAc mice and Chrimson_GCaMPBLA_NAc during the 30 min habituation session (n = 7 GCaMPBLA_NAc mice; n = 8 Chrimson_GCaMPBLA_NAc mice; unpaired t-test, p = 0.84). (C) Distance traveled was not significantly different in GCaMPBLA_NAc mice and Chrimson_GCaMPBLA_NAc during the 30 min habituation session (n = 7 GCaMPBLA_NAc mice; n = 8 Chrimson_GCaMPBLA_NAc mice, unpaired t-test, p = 0.90). All error bars are SEM. [file Image_6.JPEG]

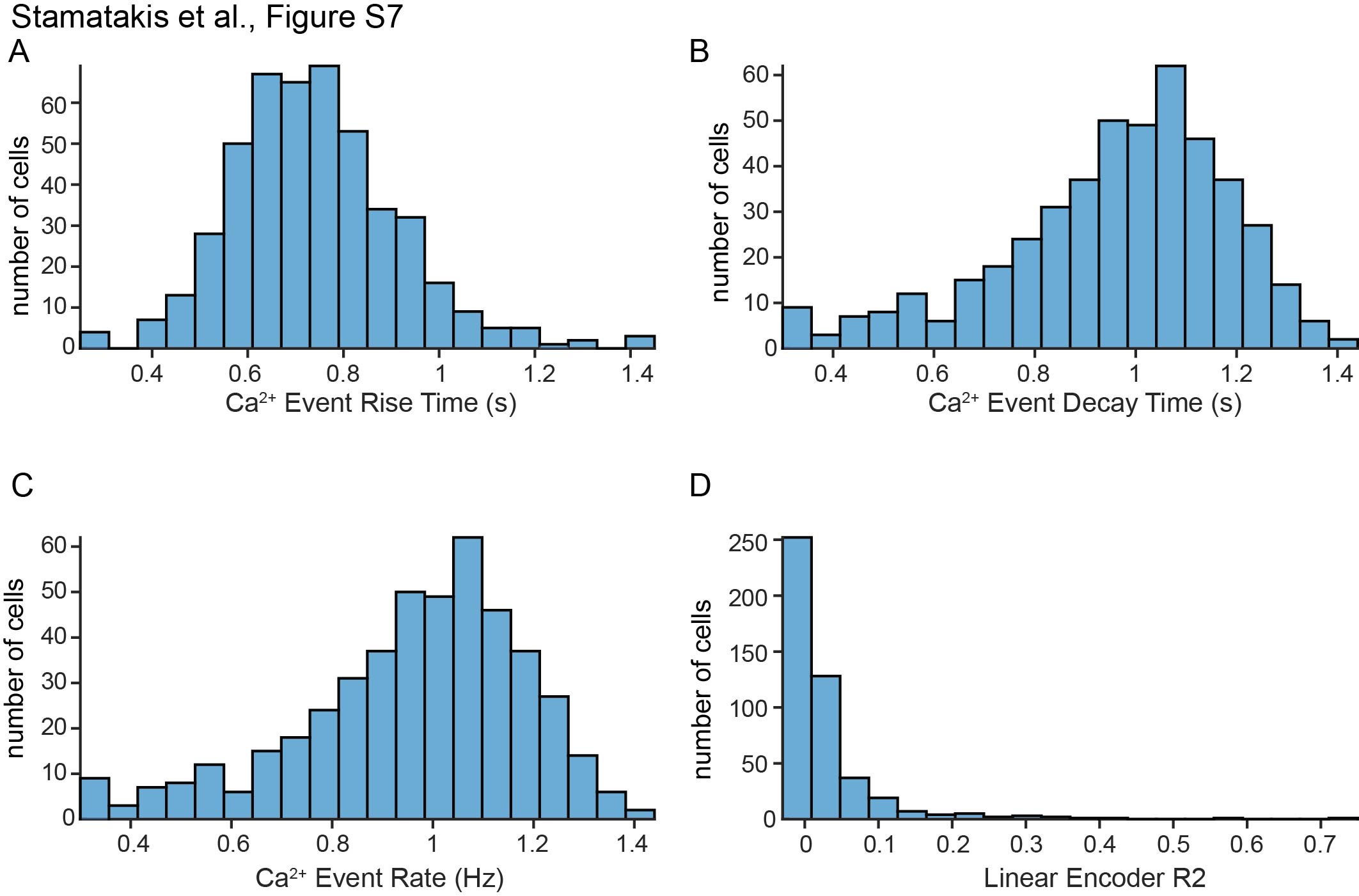

Supplement: Figure S7 — Statistics from cells included in linear filter encoder. (A) Histogram of average Ca2+ event rise times across all cells (n = 463 cells total from n = 5 GCaMPBLA_NAc mice and n = 5 Chrimson_GCaMPBLA_NAc mice). (B) Histogram of average Ca2+ event decay times across all cells (n = 463). Cells were excluded if they had an event rate of <0.01 Hz, or a decay time of <300 ms. (C) A histogram of event rates across all cells. (D) Linear filter encoder R2 values for all cells. [file Image_7.JPEG]
